# Glucose is dynamically regulated by time of day in humans and *Drosophila*

**DOI:** 10.1371/journal.pbio.3003717

**Published:** 2026-04-16

**Authors:** Dania M. Malik, Pinky Kain, Seth D. Rhoades, Arjun Sengupta, Shirley L. Zhang, Annika Barber, Paula Haynes, Erna Sif Arnardottir, Allan Pack, Richard G. Kibbey, Amita Sehgal, Aalim M. Weljie

**Affiliations:** 1 Pharmacology Graduate Group, University of Pennsylvania, Perelman School of Medicine, Philadelphia, Pennsylvania, United States of America; 2 Department of Systems Pharmacology and Translational Therapeutics, University of Pennsylvania, Perelman School of Medicine, Philadelphia, Pennsylvania, United States of America; 3 Institute for Translational Medicine and Therapeutics, University of Pennsylvania, Perelman School of Medicine, Philadelphia, Pennsylvania, United States of America; 4 Chronobiology and Sleep Institute, University of Pennsylvania, Perelman School of Medicine, Philadelphia, Pennsylvania, United States of America; 5 Fulgens Consulting, LLC, Cambridge, Massachusetts, United States of America; 6 Howard Hughes Medical Institute, University of Pennsylvania Perelman School of Medicine, Philadelphia, Pennsylvania, United States of America; 7 Department of Cell Biology, Emory University School of Medicine, Atlanta, Georgia, United States of America; 8 Waksman Institute and Department of Molecular Biology and Biochemistry, Rutgers, The State University of New Jersey, New Brunswick, New Jersey, United States of America; 9 Division of Sleep Medicine, Department of Medicine, University of Pennsylvania, Philadelphia, Pennsylvania, United States of America; 10 Center for Sleep and Circadian Neurobiology, Perelman School of Medicine, University of Pennsylvania, Philadelphia, Pennsylvania, United States of America; 11 Department of Internal Medicine, Department of Cellular & Molecular Physiology, Yale University School of Medicine, New Haven, Connecticut, United States of America; Fundacion Instituto Leloir, ARGENTINA

## Abstract

Biological clocks shape metabolism, but how circadian programs govern nutrient processing is unclear. Here, using human metabolomics and ^13^C_6_-glucose tracing in *Drosophila*, we delineate previously under characterized daily oscillations in glucose-derived metabolic networks, providing a mechanistic framework for a purpose-built isotope-tracing approach. In flies, we reveal a pronounced “rush hour” of glucose utilization early in the light phase, with carbons directed to biosynthetic and energetic pathways. By contrast, a dopamine reuptake-deficient hyperactive mutant (*fumin*) with elevated metabolic rate shows phase-shifted and amplified metabolic peaks, indicating that altered neural signaling reshapes temporal glucose flux. Neither altered feeding schedules nor short-term fasting disrupt these intrinsic metabolic rhythms, strongly suggesting that circadian timing, rather than nutrient availability, orchestrates temporal homeostasis. By integrating human metabolite profiling with isotope-tracing in flies, we define a conserved temporal architecture of glucose utilization and demonstrate that metabolic flux is dynamically gated across the day. Our findings establish a framework for understanding how circadian misalignment contributes to metabolic dysfunction and disease.

## Abstract 2

Circadian clocks are known to regulate metabolism, but the details of how they regulate glucose processing remains unclear. By integrating human metabolite profiling with isotope-tracing in Drosophila, this study defines daily rhythms in glucose utilization that are influenced by circadian timing.

## Introduction

Rhythmicity is an ever-present feature of physiology driven by zeitgebers (environmental cues), including light and temperature, that entrain endogenous circadian clocks. Molecular circadian clocks coordinate biological rhythmicity within each tissue, while modern pressures such as mistimed light exposure, shift work, and social jetlag disrupt the evolved circadian system [1–3]. Mistimed clocks play a role for increasing risk of diseases including obesity, cardiovascular disease, and cancer [4–8]. Recent molecular studies of human biofluids have shown strong rhythmicity of transcripts and metabolite levels under strict laboratory-controlled conditions [9,10]. In reality, humans vary in chronotype and display inter-individual variation in the number of rhythmic compounds and related measures (phases and amplitudes) [11–14]. In spite of this variation, certain metabolic phenomena, such as the diurnal response to nutrient intake, are relatively robust and reproducible. Although circadian regulation of glucose tolerance is well documented, the metabolic mechanisms downstream of glucose uptake—how, when, and via which pathways glucose is processed—remain poorly defined. Glucose-related measurements such as glucose tolerance and post-feeding have shown time variation with higher sugar levels reported in the afternoon versus morning measurements [15–17] (so-called “afternoon diabetes”). Moreover, afternoon measurements have been noted to allow or exclude diagnoses of diabetes where morning measurements are borderline [15]. In spite of this clinical significance, the mechanistic pathways downstream of glucose metabolism that underlie these temporal differences in glucose processing remain largely unknown.

At the molecular level, model organisms play an important role in the study of metabolic rhythmicity [18–21]. They provide the advantage of providing isogenic genetic backgrounds for study and a number of studies probing clock protein function [22] and zeitgebers such as light and feeding [23–25] have provided valuable insight into steady state metabolic pool changes at specific points in time. Our recent study [26] using an ion-switching LC-MS/MS. MS method has shown altered metabolism during the dark period in *Drosophila* short sleep mutants. However, since metabolism occurs in a network of interacting pathways, changes in the level of a given metabolite can arise from multiple possibilities and mechanistic interpretation is, by necessity, only inferred in studies where only metabolite pools are measured. Metabolic tracing provides a direct avenue to address the mechanism by which altered metabolite levels arise [27]. To date, information on diurnal rhythmic metabolic function via tracing is remarkedly sparse. Isotope tracer studies are used for clinical studies of glucose metabolism, but the fate of metabolites downstream is not known. We have recently demonstrated rhythmicity of pool sizes for glucose and related metabolites (glucose-6-phosphate and ribose-5-phosphate) in human red blood cells [28]. The further use of labeled glucose allowed for insight into rhythmicity driven specifically by glycolytic pathways and the pentose phosphate pathway noting opposite phases [28]. Other studies have also used stable and/or radioactive isotope tracers in conjunction with targeted approaches; however, even when circadian context was considered, limited number of time points were measured [29–32] so while important insight into some time-dependent differences can be ascertained, higher time resolution data are needed for reliable detection of rhythmicity [18,33].

In this study, we examined the time-of-day variability of plasma metabolites in pathways downstream of glucose metabolism in healthy humans. Based on this set of conserved pathways, which are largely conserved across species, we developed a customized analytical platform to identify physiological metabolite homeostatic rhythms in *Drosophila* via isotope tracing of glucose. We uncovered a robust, dawn-focused “rush hour” when glucose is funneled into multiple biosynthetic pathways. A number of rhythmic downstream glucose products were observed in both WT *Drosophila* as well as a hyperactive mutant with altered metabolic rate, *fumin* (*fmn*) [34], with peaks in biosynthesis from glucose during the light phase which provides direct evidence for glucose processing limitation later in the day. Together, our findings define a mechanistic framework for diurnal glucose flux and highlight the temporal plasticity of metabolic homeostasis. By demonstrating that feeding is not the sole driver of these rhythms and showing how hyperactivity modifies circadian metabolic patterns, we establish a new platform to probe the fundamental links between clock, metabolism, and disease. Understanding these relationships will be critical for developing interventions—timed diets, pharmacotherapy, or lifestyle modifications—to restore or optimize metabolic homeostasis in the face of modern circadian challenges.

## Results

### Total metabolite pools reveal time of day variation in glucose-related metabolites in humans

**Fig 1:**
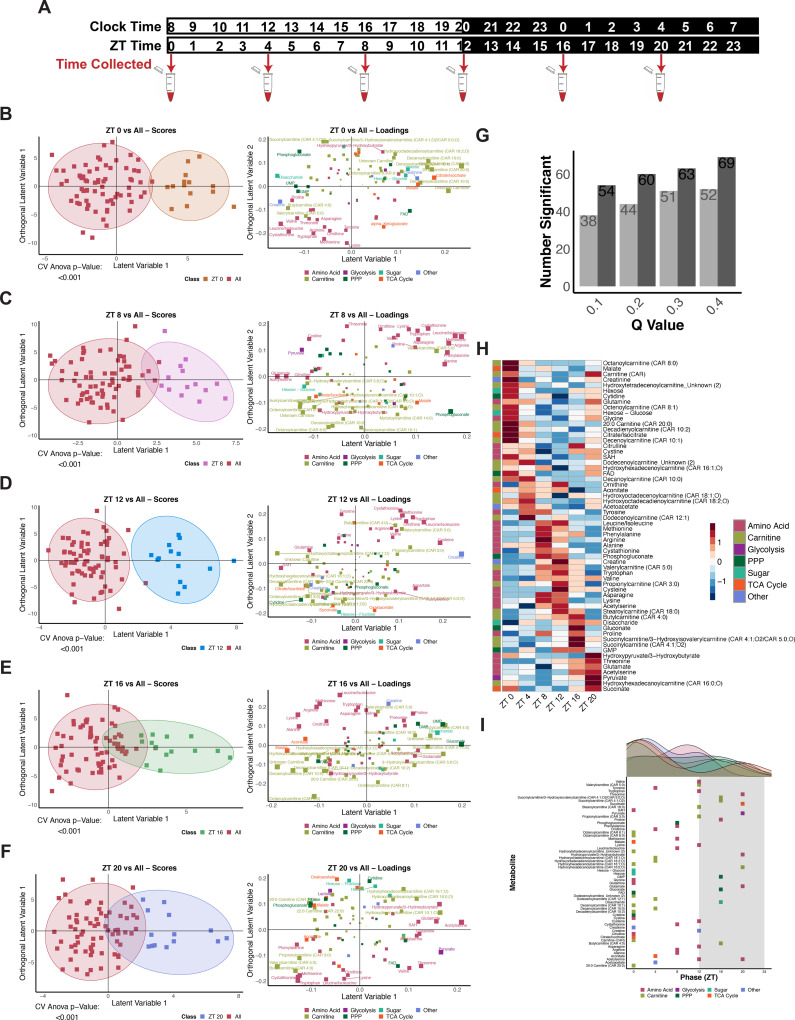
Steady state metabolite levels in human plasma samples reveal time of day variation in glucose-related metabolites. **(A)** Scores plot for the significant Time OPLS-DA Model. Classes were defined as time points (ZT 0, ZT 4, ZT 8, ZT 12, ZT 16, and ZT 20). Colors represent classes (ZT times), squares represent individual samples and triangles represent the group average. **(B)** Corresponding loadings plot for the Time OPLS-DA Model. Colors represent classes of metabolites and size represents significance as defined through VIP values. **(C)** Negative log CV-ANOVA *p*-values for overall time and each time point tested in pairwise OPLS-DA models. Significance was defined as a CV-ANOVA *p*-value less than 0.1 (shown as points above the green line). **(D)** Negative log FDR values for pathways significant in at least one significant OPLS-DA model (defined in C). Size represents impact and significance was defined as an FDR value less than 0.05. **(E)** Overview of the number of significant 24-hour rhythmic metabolites observed at RAIN (dark) or JTK (light) *q*-value cut-offs of 0.1, 0.2, 0.3, and 0.4. **(F)** Phase-ordered heatmap of significantly cycling metabolites with 24-hour periods as tested by RAIN with a *q*-value less than 0.2. **(G)** Distribution of RAIN phases for significant 24-hour metabolites. Colors represent classes of metabolites. Raw data for these figures is available as S1 Data file.

Glucose metabolism is tightly regulated by circadian processes and disruption results in various disorders, thus we first looked to establish the extent and robustness of time-of-day variability in a network of glucose-related metabolites using steady state measurements in humans. A curated set of 93 metabolites were measured in plasma samples collected every 4 hours over 24 hours (*N* = 14 subjects; S1A Fig and S1 Data) using a targeted ion-switching LC–MS method [35]. Multivariate analysis of the resulting data exhibits remarkably robust time-of-day variability (Fig 1A and S1 Data). Amino acids and related metabolites such as alanine, ornithine, cystathionine, and asparagine were noted to be elevated around zeitgeber time (ZT) 8 and ZT 12 while elevated levels of TCA cycle metabolites such as citrate/isocitrate and malate were associated with ZT 0 and 4 (Fig 1B). This result implies that the total pools of metabolites are dramatically altered throughout the day, hinting at differential pathways being available for nutrient processing. Pairwise multivariate models distinguished each single time point from a combination of the others (“one versus all”) with the exception of ZT 4. (Figs 1C and S1B–S1F). Pathway analysis of each time point model using significant metabolites demonstrates a unique set of pathways enriched at each specific time of day. For example, alanine, aspartate, and glutamate metabolism and cysteine and methionine metabolism were significantly different during the dark period; TCA cycle was significantly different throughout the day; phenylalanine, tyrosine, and tryptophan biosynthesis were significant across all of the time points, but not at a specific one (Fig 1D). Taken together the multivariate approach indicated overall differences in metabolite levels and activity of pathways at different time of the day.

Formal rhythmicity testing was performed using RAIN/JTK with Benjamini–Hochberg (BH) correction and 24-hour and 20–28-hour rhythms were observed across different *q*-value cut-offs (*q* < 0.2, 03, or 0.4, Figs 1E and S1G). For downstream analyses, we used *q* < 0.2 as a discovery-oriented threshold to balance sensitivity and specificity (expected FDR ≤20% among called rhythmic metabolites), while also reporting results across a range of *q*-value cutoffs to show robustness (Fig 1E). Metabolites with a RAIN *q*-value less than 0.2 were selected for further analysis (Figs 1F and S1H). Phases of cycling metabolites were found to be distributed across the day with a peak observed during the early light period (ZT 0–4) (Figs 1G and S1I). Taken together, these results imply that glucose tolerance is influenced by differential downstream pathways regulated in a time-of-day-dependent manner. Notably, the robustness of the response and similarity across the 14 subjects was remarkable and not obvious from individual metabolites levels. This suggests that the concerted network of glucose metabolism provides constraints under which observations of glucose metabolism should be contextualized.

### Overview and validation of isotope tracing platform

**Fig 2:**
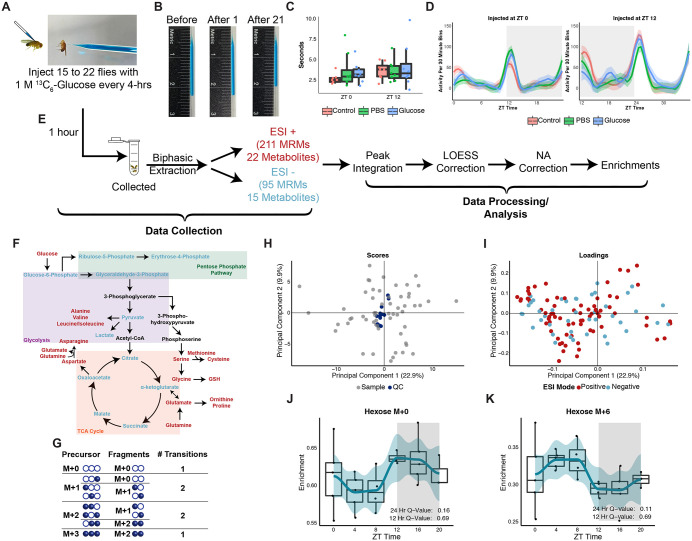
Overview and validation of isotope tracing platform and processing methods of flies injected with a labeled bolus of glucose. **(A)** Image of a fly being injected with a glucose and blue dye solution. **(B)** Representative image of a needle containing a 1 M Glucose and 0.504 mM FCF solution before injections (left), after injecting 1 fly (middle) and after injecting 21 flies (right). **(C)** climbing ability of male flies assessed through a geotaxis assay as the time needed to climb 4 cm after no injections (control) and injections with 1 M glucose or PBS. Two-sided *t* test was used with significance defined as a *p*-value less than 0.05. *n* = 5 to 8 per group and time point. **(D)** Locomotion assay of male flies without injections (control) or after injections with 1 M Glucose or PBS at ZT 0 (left panel) or at ZT 12 (right panel). Activity was recorded for 24 hours after injections. LOESS trace is shown for activity counts for each 30-minute time period. Significance was tested using a two-sided *t* test for each activity bin across groups with significance defined as a BH adjusted *p*-value (*q*-value) less than 0.05. *n* = 8 per group and time point. **(E)** Simplified overview of workflow. Flies (*n* = 15 to 23) injected with 1 M Glucose were collected 1 hour post-injection. Upper fractions (polar metabolites) collected from a biphasic extraction were analyzed in positive and negative electrospray ionization modes. Acquired data was integrated to obtain ion counts which were used for an instrumental drift correction and normalized to the amount of blue dye injected. The normalized data is natural abundance corrected and used to calculate enrichments for further analysis. **(F)** Simplified view of downstream glucose metabolism with metabolites included in MS methods highlighted in red (ESI+) and blue (ESI−). Shading indicates downstream glucose pathways. **(G)** Depiction of labeling patterns considered for generating MRM transitions of a 3-carbon compound, such as alanine, with a 2-carbon fragment. In generating transitions, the number of potential labeled carbons in the precursor and fragments are considered for each isotopologue. **(H)** PCA scores plot of biological (light gray) and QC samples (blue) showing clustering of the QC samples. **(I)** PCA loadings plot corresponding to the scores plot (H) showing the distribution of metabolite enrichments detected in positive (red) and negative (blue) modes. **(J, K)** Box plot of enrichments along with a LOESS trace of hexose M + 0 (J) and hexose M + 6 (K) observed across a day at 4 hour intervals in wild-type flies. *n* = 4 to 5 biological replicates of 15 male flies per time point. Rhythmicity of 24- and 12-hour periods was tested using RAIN and significance was defined as a BH-adjusted *q*-value less than 0.2. Raw data for these figures is available as S2 (C), S3 (D), and S4 (E–K) Data files.

The pathways measured related to glucose metabolism are largely conserved across species, and thus we developed a mass spectrometry-based platform in *Drosophila* to trace the fate of glucose metabolites through a broad network of downstream metabolism, as observed in the human study above. The use of isotope tracers such as uniformly labeled ^13^C_6_-glucose enable the metabolites of interest (downstream of glucose) to be monitored at the atomic level, thus allowing further insights into how glycemic metabolism is altered as a function of time. Uniformly labeled ^13^C_6_-glucose was chosen as the stable isotope tracer as it allows carbon flow to be monitored into downstream metabolites spanning the TCA cycle, pentose phosphate pathway (PPP), glycolysis, and amino acid biosynthesis. A range of glucose concentrations were tested in *Drosophila* to identify an optimal concentration at which *Drosophila* remained viable. Flies were sampled one hour after introducing the tracer to allow glucose to be metabolized into downstream pathways and to maintain sufficient time resolution to monitor diurnal shifts. The 1 M bolus of glucose was introduced intra-thoracically using a blue dye as a visual aid. The injections were performed on flies that were sedated with cold treatment to prevent metabolic changes caused by hypoxia due to carbon dioxide exposure (Fig 2A and 2B). Representative images of a needle containing the injection solution highlight the small volume (~31 nl/fly) introduced into flies (Fig 2B). Since a non-physiological glucose concentration (~25.6 µM/fly corresponding to ~0.8 pmol/fly or ~148 pg/fly) was used to allow for detection by mass spectrometry, geotaxis and locomotion assays were used to assess whether the glucose and/or blue dye were impacting physiology (S2 and S3 Data files). No significant differences were noted between the glucose and PBS injected conditions for both climbing ability and activity (Fig 2C and 2D). This indicated that the supraphysiological glucose and dye concentrations did not impair geotaxis or locomotion of WT flies and thus were chosen as a tracer to assess time-resolved metabolic nutrient challenge.

We challenged WT *Drosophila* with 1 M uniformly labeled ^13^C_6_-glucose at 4-hour intervals across a day to determine how glucose is processed at different times in the fly body (Fig 2E and S4 Data). Each sample was analyzed using two LC–MS methods covering 22 and 15 downstream glucose metabolites in electrospray ionization positive (211 transitions) and negative (95 transitions) modes, respectively (Fig 2E and 2F). Metabolite transitions were defined by considering the number of carbons and possible labeling patterns of the precursor and fragment (Fig 2G). The resulting data were corrected for instrumental drift and normalized to the blue dye ion counts to adjust for variability in injection volumes before performing a natural abundance (NA) correction. Enrichments were calculated using the NA-corrected data and were used for further analyses (Fig 2E).

To determine data quality, enrichments for all biological and quality control (QC) samples were visualized using a PCA (Fig 2H and 2I). Overall analytical quality was robust as noted through clustering of the QC samples in the scores plot (Fig 2H) and evenly distributed compounds from both ionization modes in the loadings plot (Fig 2I). Furthermore, the platform was sensitive to detect changes in levels of detected isotopologues (chemicals with the same chemical formula but different isotopic compositions) one hour after samples were challenged with labeled glucose at 4-hour intervals. For example, opposing changes were noted in the enrichments of two hexose isotopologues, M + 0 and M + 6 (Fig 2J and 2K and S4 Data). Here, hexose M + 0 (composed primarily of glucose) peaked around ZT 12 (lights off) while the M + 6 (composed of all labeled glucose) form peaked around ZT 4. This points to potential changes in overall glucose usage during the day.

### Increased biosynthesis from glucose challenge is distributed across multiple pathways at ZT 4

**Fig 3:**
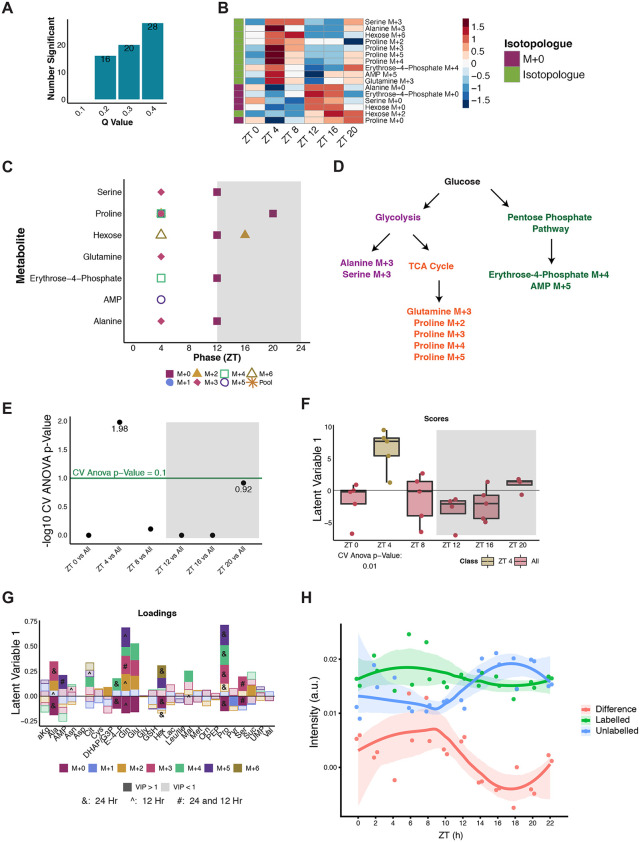
Increased biosynthesis from glucose challenge is distributed across multiple pathways at ZT 4. **(A)** Overview of the number of significant 24-hour rhythmic compounds observed at RAIN *q*-values of 0.1, 0.2, 0.3, and 0.4. **(B)** Phase-ordered heatmaps of significantly cycling compounds with 24-hour periods in WT as tested by RAIN with a *q*-value less than 0.2. **(C)** Distribution of RAIN phases for significant 24-hour compounds grouped by metabolite. Shapes and colors refer to isotopologues or pools. **(D)** A simplified overview of downstream metabolism indicating potential routes for the biosynthesis of 24-hour rhythmic isotopologues from B. **(E)** Negative log CV-ANOVA *p*-values for each time point tested in pairwise OPLS-DA models. Significance was defined as a CV-ANOVA *p*-value less than 0.1 (shown as points above the green line. **(F)** Scores plot for the significant WT ZT 4 vs. All OPLS-DA Model. Two classes were defined as ZT 4 and All (ZT 0, ZT 8, ZT 12, ZT 16, and ZT 20). **(G)** Corresponding loadings plot for the WT ZT 4 vs. All OPLS-DA model. Colors represent isotopologues with shading representing VIP significance (dark signifies a VIP value greater than 1 (significantly contributing to separation of ZT 4 from other time points) while lighter shades are used for a VIP less than 1). Rhythmic isotopologues from S2 Fig is indicated by symbols (& refers to significant 24-hour rhythmicity only, ^ refers to significant 12-hour rhythmicity only, # refers to significant 24- and 12-hour rhythmicity). alpha-ketoglutarate (a-kG); alanine (Ala); adenine monophosphate (AMP); asparagine (Asn); aspartate (Asp); citrate (Cit); cysteine (Cys); dihydroxyacetone phosphate (DHAP); glyceraldehyde-3-phosphate (G3P); erythrose-4-phosphate (E-4-P); glutamine (Gln); glutamate (Glu); glycine (Gly); glutathione (GSH); hexose (hex); lactate (Lac); leucine (Leu); isoleucine (Ile); malate (Mal); methionine (Met); ornithine (Orn); phosphoenolpyruvate (PEP); proline (Pro); serine (Ser); succinate (Suc); uridine monophosphate (UMP); valine (Val). **(H)** NMR-derived lipid quantification of ROI2 from lipid extracts of ^12^C and U-^13^C-glucose injected flies. The difference represents the net ^13^C incorporation into the total detected lipid pool for a specific class of lipid methyl groups. Raw data for these figures is available as S4 and S5 Data files.

To further understand changes in glucose utilization as a function of time in WT *Drosophila*, JTK and RAIN algorithms were used to test for 24-, 20−28-, and 12-hour rhythmicity. As the FDR (*q*-value) cut-off was increased from 0.1 to 0.4, increasing numbers of isotopologues cycled with periods of 24-, 20−28-, and 12-hour periods (Figs 3A, S2A, and S2D and S4 Data). For further analyses, rhythmic compounds with an FDR less than 0.2 were used (Figs 3B, S2B, and S2E and S4 Data). For all periods tested, metabolite pools (sum of all isotopologues for a given metabolite) were not significantly rhythmic. For 24- and 20–28-hour rhythmic results, labeled isotopologues peaked at ZT 4 while unlabeled (M + 0) isotopologues peaked at the light:dark (LD) transition (ZT 12) (Figs 3C and S2C and S4 Data) which is to be expected based on the hexose labeling pattern. On the other hand, 12-hour rhythmic isotopologues peaked mostly around ZT 8 and ZT 20 (S2F Fig). Since 24-hour rhythmicity was not present in metabolite pools, this suggests labeled glucose carbons were incorporated into isotopologues such as serine M + 3, proline M + 2, M + 3, M + 4, and M + 5 at ZT 4. Taken together, peaks at ZT 4 for erythrose-4-phosphate M + 4 and AMP M + 5 can imply use of the PPP while M + 3 forms of serine and alanine may arise downstream of glycolysis, and glutamine and proline isotopologues may arise from the TCA cycle (Fig 3D). This points to overall increased activity of downstream glucose metabolic pathways uniquely at ZT 4, which we will refer to as a “rush hour” of biosynthesis.

To determine if time-of-day differences in glucose usage persisted at an overall metabolic profile level, a multivariate approach using orthogonal partial least squares-discriminant analysis (OPLS-DA) models was employed to determine whether any time point was unique from the rest. From all time points tested, ZT 4 emerged as a distinct time point compared to all remaining time points (Fig 3E and 3F and S4 Data). Higher enrichments of isotopologues such as alanine M + 2, glutamine M + 2 to M + 5, glutamate M + 2 to M + 4, and proline M + 3 to M + 5 and lower enrichments of M + 0 forms (alanine, glutamine, glutamate, and proline) were associated with ZT 4 (Fig 3G and S4 Data). This further supported the rhythmicity analysis in highlighting increased incorporation of labeled glucose into downstream products at ZT 4 suggesting increased biosynthesis.

Because circadian structure in small-molecule pools can reflect insect-specific redox shuttling (e.g., the proline–alanine cycle) rather than net anabolism, we sought orthogonal evidence for biosynthetic allocation into larger molecular pools. We therefore injected flies with either unlabeled glucose or [U-^13^C]-glucose and acquired ^1^H–^13^C HSQC spectra (S3A and S3B Fig and S5 Data). Using ROI definitions summarized in the HSQC ROI heatmap (S3B Fig), we quantified lipid-associated regions; ROI2 (S3A Fig) corresponds to cross-peaks in the aliphatic lipid region consistent with lipid-associated methyl groups. Labeling kinetics were temporally structured. In contrast to the early “rush hour” of ^13^C in small-molecule metabolites around ZT 4, consistent with rapid glucose entry into central metabolism, the lipid-associated HSQC ROIs showed a delayed/shifted pattern. For ROI2, the labeled–unlabeled difference (net ^13^C incorporation into this lipid-associated ROI) peaked in the mid-to-late light time (~CT6–10) (Fig 3H and S5 Data) and reached a minimum later in the dark period (~CT16–20), consistent with time-of-day variation in allocation of glucose-derived carbon into lipid-associated pools. We therefore interpret the temporal program as comprising both central-metabolism flux changes (which may include proline–alanine cycling) and a measurable component of biosynthetic allocation into lipid-associated carbon.

### Temporal-gating of glucose processing is dependent on the circadian clock

To determine whether the observed “rush hour” of glucose flux at ZT 4 is driven by the endogenous circadian clock, we performed U-^13^C₆-glucose tracing in the arrhythmic period null mutant, *per⁰¹.* These mutants lack a functional molecular clock, allowing us to disentangle clock-driven regulation from passive responses to environmental cues like light. Following the same time-resolved injection protocol used for wild-type flies, we quantified the labeling fraction of glucose isotopologues (*f*_labeled_ = 1 − (M + 0/∑_*i*_M + *i*) (S3C Fig and S6 Data). The analysis revealed a complete loss of rhythmic glucose processing in the absence of a functional clock. While wild-type flies showed a robust 24-hour rhythm in hexose M + 6 labeling (RAIN, *p* = 0.03), this rhythm was entirely abolished in *per⁰¹* mutants (RAIN, *p* = 0.9). As illustrated in S3C Fig, the diurnal oscillation in glucose incorporation observed in wild-type flies is attenuated in *per⁰¹* mutants, which exhibit an ultradian-like labeling pattern across the day. This result provides direct evidence that the temporal gating of glucose into central carbon metabolism is actively regulated by the core molecular clock.

### The TCA Cycle is preferentially used in a hyperactive mutant in response to a glucose challenge

**Fig 4:**
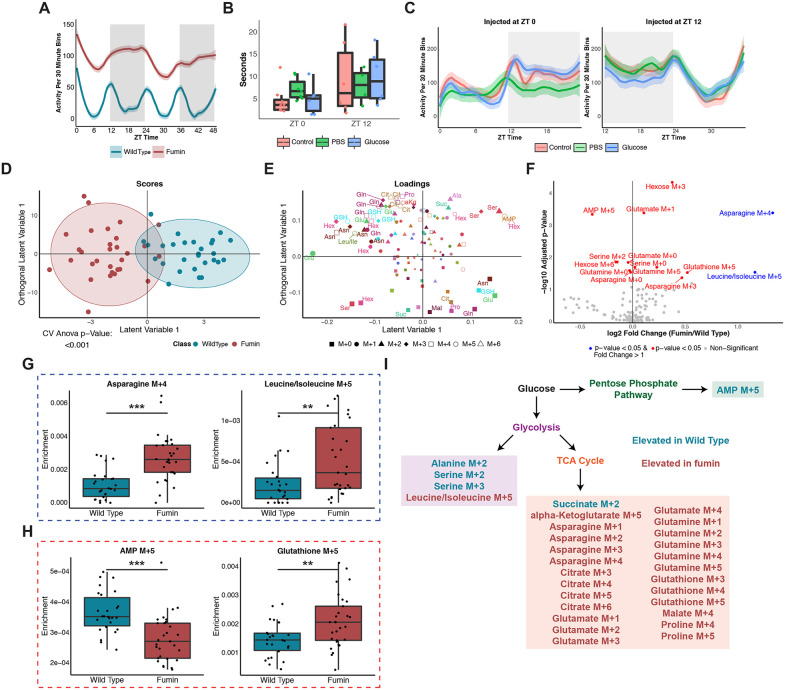
The TCA Cycle is preferentially used in a hyperactive mutant in response to a glucose challenge. **(A)** LOESS trace of activity counts per 30 min bins for WT and *fmn* over 48 hr under 12-hour LD conditions. **(B)** Climbing ability of male *fmn* flies assessed through a geotaxis assay as described in Fig 2C. **(C)** Locomotion assay of male *fmn* flies assessed through a locomotion assay as described in Fig 2D. **(D)** A two-component OPLS-DA scores plot of WT vs. *fmn*. All time points collected (ZT 0, 4, 8, 12, 16, and 20) are included for both classes defined as genotypes. *n* = 4 to 5 biological replicates with 15 male flies per time point and genotype. **(E)** Corresponding loadings plot for the WT vs. *fmn* OPLS-DA model (D). Isotopologues are represented by shapes, metabolites by color and size of the shapes represents significance as defined through VIP values. Abbreviations as defined in Fig 3G. **(F)** Volcano plot of enrichments analyzed through univariate analyses. log2 of the fold change between *fmn* and WT is shown on the x-axis while the negative log10 BH-adjusted genotype *p*-value from a two-way ANOVA is shown on the y-axis. A fold change cutoff of greater than 1 was used and a *p*-value less than 0.05 was defined as significant. **(G, H)** Representative boxplots of enrichments by genotype for significant isotopologues by *p*-value and fold change (G) or by *p*-value only (H). **(I)** A simplified overview of downstream glucose metabolism indicating potential routes for the biosynthesis of VIP metabolites from E. Isotopologue text color refers to the genotype with higher loadings. Raw data for these figures is available as S2 (B), S3 (A and C), and S4 Data files.

As activity levels can act as a weak zeitgeber to influence the clock [36], we next sought to determine how glucose is used differently in a hyperactive short-sleeping mutant deficient in dopamine reuptake, *fumin* (*fmn*) [34]. The *fmn* mutation leads to a defective dopamine transporter resulting an increased amount of dopamine signaling in the synapse. Consistent with the critical and conserved role of dopamine system in regulating arousal and sleep, *fmn* flies exhibit reduced sleep and prolonged activity compared to wild-type flies. The increase in activity is accompanied by an up-regulated metabolic rate, making the *fmn* mutant a vital tool to provide unique metabolic fingerprints. Overall, *fmn* displays elevated activity profiles as compared to WT with higher levels observed during the dark period (Fig 4A and S3 Data). We challenged the mutant with glucose in a similar manner to WT. The climbing ability and activity of *fmn* were also not impaired in flies injected with glucose versus PBS at both ZT 0 and ZT 12 (Fig 4B and 4C; S2 and S3 Data). Therefore, the injection of supraphysiological concentrations of glucose did not dramatically alter the physiology of the flies.

Overall, wild type and *fumin* process glucose distinctly as indicated by a significant two-component discriminant (OPLS-DA) model (Fig 4D and S4 Data). Hexose M + 6 enrichments were relatively higher in WT while M + 0 enrichments were relatively higher in *fmn* suggesting that labeled glucose was more readily used in *fmn*. Overall, enrichments of primarily labeled isotopologues (i.e., glutamate M + 1, glutathione M + 5, and asparagine M + 2 and M + 4) contributed to the separation of *fmn* from WT while enrichments of several unlabeled isotopologues such as asparagine, glutamate, and glutathione were more associated with WT samples (Fig 4E). Univariate analyses of genotype differences also showed similar changes (Fig 4F). For instance, enrichments of asparagine M + 4 and leucine/isoleucine M + 5 were significantly higher (at least 2×) in *fmn* as compared to WT (Fig 4G). Since leucine and isoleucine are essential amino acids, the synthesis of these may be a byproduct of the fly microbiome. Additional significant differences such as higher AMP M + 5 levels in WT and elevated glutathione M + 5 levels in *fmn* were noted through the adjusted genotype *p*-value (Fig 4H). As glutathione M + 5 requires either glutamine M + 5, or glutamine M + 3 and glycine M + 2 as precursors, we also looked at the correlation of these isotopologues. Glutathione M + 5 enrichments were found to be significantly correlated with glutamine M + 3 (*p* < 0.05) and weakly correlated with glutamine M + 5 (*p* < 0.1) enrichments in *fmn* (S4A Fig). From both the univariate and multivariate approaches, relatively higher enrichments of AMP M + 5, a metabolite related to the PPP, and metabolites associated with glycolysis (alanine M + 2, serine M + 2 and M + 3) were observed in WT while metabolites involved in or downstream of the TCA cycle (alpha-ketoglutarate M + 5, asparagine, glutamate, and glutamine isotopologues) had relatively higher enrichments in *fmn* (Fig 4I). Overall, both multivariate and univariate approaches support increased TCA cycle labeling in *fmn* while glycolysis and PPP labeling is associated with WT.

### fumin has two biosynthetic “rush hours” at dawn and dusk

In addition to overall genotype-level differences, we sought to determine how the two genotypes differed in glucose utilization through time. Through pairwise OPLS-DA models of the genotypes at each time point, a significant model was observed at ZT 4 (S4B and S4C Fig and S4 Data). Three types of changes were noted in labeled isotopologues: 1. Higher relative enrichments in WT (i.e., alanine M + 1 to M + 3 and proline M + 3 to M + 5); 2. Higher relative enrichments in *fmn* (i.e., alpha-ketoglutarate M + 2 to M + 4, asparagine M + 1 to M + 4, and glutathione M + 1, M + 2 and M + 5); and 3. Higher enrichments spread across the two genotypes (i.e., higher AMP M + 5 and lactate M + 3 in WT versus higher AMP M + 1 and lactate M + 2 in *fmn*) (S4D Fig). This suggests differences in downstream glucose products and/or differences in glucose utilization pathways in WT when compared to *fmn*. Overall, both multivariate and univariate approaches supported the idea that glucose is differentially used between the two genotypes at ZT 4 with increased TCA cycle labeling observed in *fumin*.

**Fig 5:**
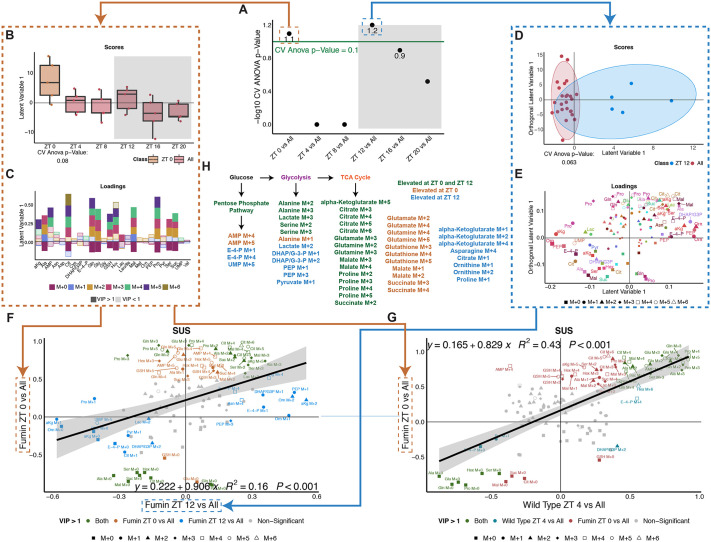
Excess glucose processing is unique at dawn and dusk compared to other times of day in *fumin.* **(A)** Negative log CV-ANOVA *p*-values for each time point tested in pairwise OPLS-DA models for *fmn*. Significance was defined as a CV-ANOVA *p*-value less than 0.1 (Shown as points above the green line). **(B)** Scores plot for significant *fmn* ZT 0 vs. All OPLS-DA model. Two classes were defined as ZT 0 and All (ZT 4, ZT 8, ZT 12, ZT 16, ZT 20). **(C)** Corresponding loadings plot for the *fmn* ZT 0 vs. All OPLS-DA model. Colors represent isotopologues with shading representing VIP significance (dark signifies a VIP value greater than 1 while lighter shades are used for a VIP less than 1). Abbreviations are defined in Fig 3G. **(D)** A two-component scores plot for the significant *fmn* ZT 12 vs. All model. Classes were defined as ZT 12 and All (ZT 0, ZT 4, ZT 8, ZT 16, and ZT 20). **(E)** Corresponding loadings plot for the *fmn* ZT 12 vs. All OPLS-DA model. Isotopologues are represented by shapes, metabolites by color and size of the shapes represents significance as defined through VIP values. Abbreviations as defined in Fig 3G. **(F)** SUS plot comparing the loading plots from the *fmn* ZT 0 vs. All (C) and the *fmn* ZT 12 vs. All OPLS-DA models (E) with a regression line and equation. Green refers to VIP compounds in both models, orange refers to VIP compounds only in the *fmn* ZT 0 vs. All model, and blue refers to VIP compounds only in the *fmn* ZT 12 vs. All model. Shapes refer to isotopologues. **(G)** SUS plot comparing the loadings plot from the *fmn* ZT 0 vs. All (C) and the WT ZT 4 vs. All (Fig 3G) OPLS-DA models with a regression line and equation. Green refers to VIP compounds in both models; blue refers to VIP compounds in the WT model; red refers to VIP compounds in the *fmn* model. Shapes refer to isotopologues. **(H)** A simplified overview of downstream glucose metabolism indicating potential routes for the biosynthesis of VIP metabolites highlighted in F according to time point. Isotopologue text color refers to time point. Raw data for these figures is available as S4 Data file.

Next, to determine whether glucose is processed differently by *fumin* based on time-of-day, pairwise discriminant models were generated to test whether a time point(s) was distinct from all other time points. Both ZT 0 and ZT 12 emerged as being significantly distinct from other time points through one- and two-component models, respectively (Fig 5A, 5B, and 5D, and S4 Data). Increased enrichments of labeled isotopologues were noted at both time points, ZT 0 and ZT 12, pointing to increased incorporation of glucose carbons into the isotopologues (Fig 5C and 5E). Furthermore, the light time points (ZT 4 and ZT 8) were significantly different from the dark ones (ZT 16 and ZT 20) in an OPLS-DA model (S5A Fig). The light time points were associated with relatively increased enrichments of labeled isotopologues (S5B Fig).

### Proline supplementation reveals a delayed, genotype-specific vulnerability in fmn under repeated glucose challenge

To probe whether proline-linked redox handling contributes to the altered metabolic state of *fmn*, we performed additional experiments using dietary proline to stress proline-linked redox buffering and asked whether this would differentially affect wild-type versus *fmn* flies across time of day. Both wild-type and *fmn* flies were exposed to 30 and 15 mM proline (S6A–S6H Fig and S7 Data) separately during the pre-feeding phase (5 days) and on day 6 (day 0 on graphs), flies were challenged with 1 M ^12^C_6_-glucose at ZT 4 and ZT 16 (injected group; Figs S6B, S6D, S6F, and S6H). First, to rule out trivial intake or baseline toxicity effects, we quantified survival of flies on 30 and 15 mM proline food during the pre-feeding phase. *fmn* mutants exhibited lethality (29% to 46%) during the pre-feeding phase to 30 mM but not to 15 mM proline, whereas wild-type flies tolerated proline feeding well. All the flies that survived during the pre-feeding phase were grouped on day 6 into 3 sets each (control group; Figs S6A, S6C, S6E, and S6G) and (injected group) with 20–25 flies each set for ZT 4 and ZT 16. For control group, flies were transferred to fresh proline media on Day 6 (15 or 30 mM). We observed no significant difference in lethality between wild-type and *fmn* flies at ZT 16 (15 or 30 mM proline control condition), indicating that both genotypes survived well under control conditions at this time point. Notably, we did not observe significant differences in lethality in flies injected with ^12^C_6_-glucose at ZT 4, but *fmn* flies maintained on 30 mM proline exhibited clear lethality on days 2–3 at ZT 16 in the injected group, whereas wild-type flies on the same proline-supplemented medium remained largely viable over the same time frame. For proline 15 mM condition, no differences in survival were noticed in general, only wild-type flies showed some lethality at day 2 in ZT 4 injected group. Thus, proline loading selectively compromised survival in *fmn* in the context of repeated glucose challenges, revealing a delayed vulnerability consistent with impaired redox-buffering capacity in the mutant.

**Fig 6:**
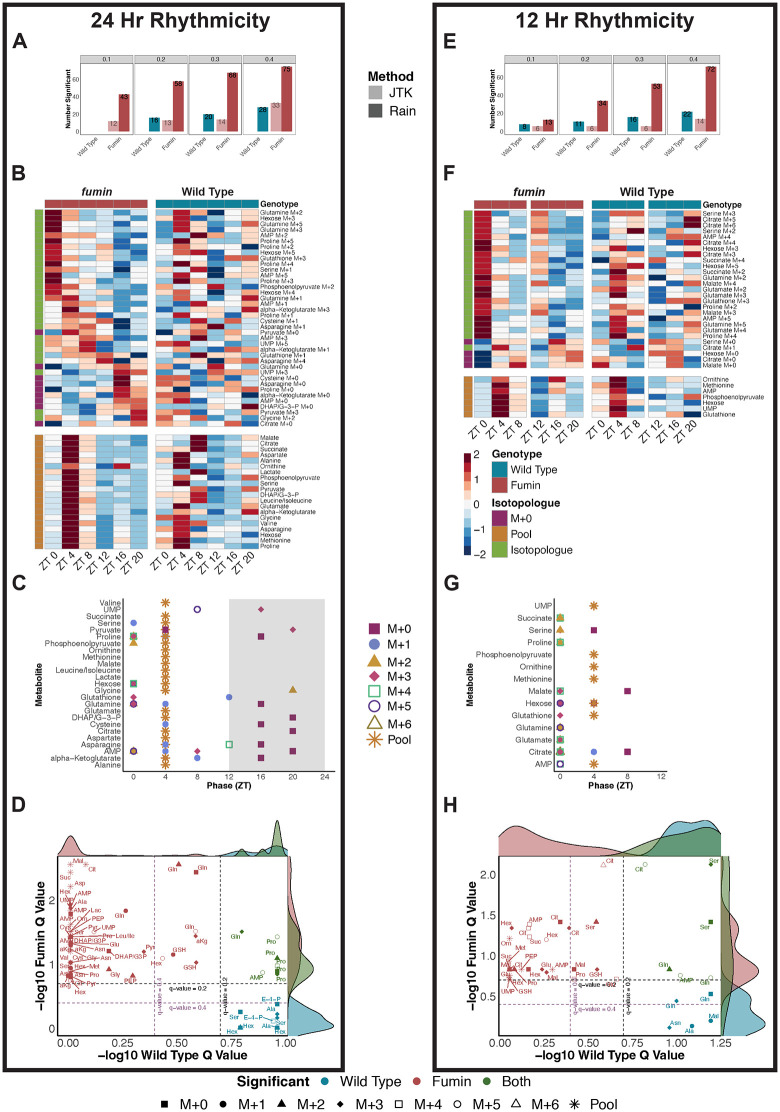
Increased diurnal and ultradian biosynthetic rhythms in *fumin.* **(A, E)** Overview of the number of significant 24-hour (A) and 12-hour (E) rhythmic compounds observed at RAIN (dark shade, depicted with dark gray) or JTK (light shade) *q*-value cut-offs of 0.1, 0.2, 0.3, and 0.4. **(B, F)** Phase-ordered heatmaps of significantly cycling compounds with periods of 24-hour (B) or 12-hour (F) in *fmn* at a RAIN *q*-value cutoff of 0.2. Enrichments for WT are shown for comparison. Legend for both heatmaps is shown with F. **(C, G)** Distribution of RAIN phases for significant 24-hour (C) and 12-hour (G) compounds in *fmn* grouped by metabolite. Shapes and colors refer to isotopologues or pools. **(D, H)** Comparison of 24-hour (D) or 12-hour (H) significant cycling compounds in WT and *fmn* as defined by a RAIN *q*-value less than 0.2. *q*-value cutoffs of 0.2 (black dotted line) and 0.4 (purple dotted line) are shown. Color refers to significant rhythmicity in one or both genotypes while shapes refer to isotopologues. Raw data for these figures is available as S4 Data file.

### Only the dawn biosynthetic “ush hour” is similar to WT “rush hour”

Given that *fmn* had two peaks with strong biosynthetic activity, we then investigated whether excess glucose metabolism at ZT 0 and ZT 12 followed a similar set of pathways in *fmn* using a shared and unique structures (SUS) plot (Fig 5F and S4 Data). In this analysis, the loadings (or distribution of isotopologue enrichments) from both models were plotted against each other with ZT 0 versus All (Fig 5C) on the y-axis and ZT 12 versus All (Fig 5E) on the x-axis. The resulting plot highlighted a weak correlation between the two-time points through both unique (blue and orange), as well as conserved (green) metabolism (Fig 5F). For example, enrichments of ornithine M + 1 and alpha-ketoglutarate M + 2 were relatively higher at both ZT 0 and ZT 12, but were only significantly contributing to the separation of ZT 12 from other time points. On the other hand, enrichments of isotopologues such as glutamate M + 2 and M + 4 only significantly contributed to the separation of the ZT 0 time point. From both models, broad labeling of downstream glucose products with differences in metabolite and/or labeling were noted at both time points. Overall increased TCA cycle labeling was apparent at both time points through unique and common isotopologues while glucose carbons may be incorporated into glycolysis more at ZT 12 (Fig 5H). Overall, this highlights that although a few similar pathways may be activated in *fmn* at the two LD transitions (or dawn and dusk), generally different isotopologues are generated (i.e., unique metabolic pathways used).

Since *fumin* at ZT 0 and ZT 12 and wild type at beginning of the light phase (ZT 4) had all shown increased biosynthesis from labeled glucose, we next looked to determine whether *fmn* used glucose similarly to WT at these time points. When comparing the loading plots from the early light period from both models (e.g., WT ZT 4 versus All (Fig 3G) and the *fmn* ZT 0 versus All (Fig 5C), the resulting SUS model highlighted a notable degree of correlation between the two conditions (Fig 5G). Furthermore, several conserved compounds significantly contributed to the separation of both ZT 4 in WT and ZT 0 in *fmn*, highlighted in green. For example, proline M + 4 and glutamine M + 3, M + 4, and M + 5 enrichments were relatively higher at both ZT 4 in WT and ZT 0 in *fmn* relative to other time points*.* On the other hand, compounds significantly contributing to the separation of *fmn* ZT 0 (VIP compounds such as citrate M + 3, M + 5, and M + 6) were also noted and although they displayed similar changes in WT, they did not meet the significance cutoff as defined by a VIP value greater than one. In contrast, fewer concerted changes were observed between *fmn* at ZT 12 and WT at ZT 4 (S5C Fig). This analysis suggests that there is a similar and specific set of metabolic pathways activated at ZT 4 in WT and ZT 0 in *fmn* compared to other timepoints indicating a potential phase advancement in *fmn* of metabolic pathways primed to utilize glucose in the early morning period.

### Glycogen-associated labeling is genotype dependent

To test whether circadian variation in glycogen buffering could contribute to the bolus tracer phenotype, we performed two independent glycogen-focused assays: (1) whole-body extracts were enzymatically hydrolyzed to convert glycogen to glucose prior to isotopologue quantification of the hexose M + 6/M + 0 ratio across circadian time (S7A Fig and S8 Data), and (2) total glycogen pools were measured (S7B–S7D Fig and S9 Data). WT total glycogen/protein was comparatively stable across ZT (RAIN *p* = ns), whereas *fmn* showed markedly reduced glycogen with time-of-day structure (RAIN *p* = 1.73 × 10⁻⁵; amplitude ≈ 0.17), including a pronounced reduction near early day (S7A Fig). The hydrolysis-derived hexose labeling metric (M + 6/M + 0) exhibited stronger circadian structure in *fmn* than in the control background, indicating genotype-dependent temporal regulation of glycogen-associated labeling. Together, these data indicate that time-dependent glycogen-associated labeling is not a dominant feature of baseline glucose handling in the control background, but becomes strongly time-structured in the hyperactive, high-energy-demand *fmn* genotype.

### Increased diurnal and ultradian biosynthetic rhythms in fumin

Having observed unique processing of glucose at two time points in *fumin*, we next asked if the changes are due to underlying cycling over the course of a day. To address isotopologue rhythmicity, 20–28-, 24-, and 12-hour periods were tested. Overall, a greater number of rhythmic isotopologue enrichments and/or pools were observed in *fmn* than WT for all periods tested across different RAIN and JTK FDR cut-offs (Figs 6A, 6E, S8A, and S4 Data). For further analyses, compounds meeting a 0.2 RAIN FDR cut-off were used (Figs 6B, 6F, and S8B). In contrast to WT, metabolite pools cycled with 20–28-, 24- and 12-hour periods. Most of the significant 24-hour cycling labeled isotopologue enrichments were noted to peak around ZT 0 to ZT 4 while unlabeled forms peaked during the dark period between ZT 16 and ZT 20 and pools were noted to peak around ZT 4 (Fig 6C). Since the peak in overall metabolite levels (pools) and enrichments (labeled isotopologues) both occurred between ZT 0 and ZT 4, this suggested that these arose from the incorporation of labeled glucose. However, alternative sources of carbon contribution cannot be ruled out. This trend was also observed in phases of significant 20–28-hour cyclers (S8C Fig). Similar to the observed 24-hour rhythmicity, significant compounds with 12-hour periods also showed a number of labeled isotopologues with peaks between ZT 0 and ZT 4 along with pools peaking at ZT 4 and non-labeled forms, M + 0, peaking around ZT 8 (Fig 6G). In this case, because pools are peaking in between labeled and unlabeled forms, it is difficult to determine whether overall pool levels are increased due to biosynthesis from glucose. However, it is likely that glucose carbons contributed to the increased pool levels noted as alternative routes are limited. Overall, rhythmicity analyses support increased biosynthesis of isotopologues at the LD transitions in *fmn* with a substantial contribution to the increased biomass arising from glucose carbons. Furthermore, the peak of rhythmicity around ZT 0 in *fmn* also supports the idea of phase-advanced biosynthesis from WT.

Since rhythmicity was observed in both genotypes, we next explored whether similar compounds were cycling in WT and *fmn*. For 24-hour rhythmic compounds, about 50% of the isotopologues cycling in WT were also noted to be 24-hour rhythmic in *fmn* (Fig 6D). A similar observation was also noted with 20–28 hour rhythmic compounds (S8D Fig). In contrast, *fmn* primarily contained unique isotopologues and/or pools that were rhythmic, but it is important to note that a few are trending towards significance in WT (glutathione M + 1 and M + 3 and glutamine M + 0 and M + 2). A similar trend was also noted for 12-hour rhythmic compounds (Fig 6H). However, glutamine M + 0 and M + 3 were significant in WT for 12-hour periods but trended towards significance in *fmn*. This highlights that a majority of the cycling observed in *fmn* was uniquely gained rather than conserved or lost from the cycling observed in WT. Overall, rhythmicity analyses also supported TCA cycle labeling in *fumin* with contributions from both diurnal and ultradian rhythmicity while cycling of glycolysis and PPP related isotopologues was primarily diurnal.

### Shift from ultradian to diurnal rhythmicity is observed in product to precursor relationships in fumin compared to wild type

**Fig 7:**
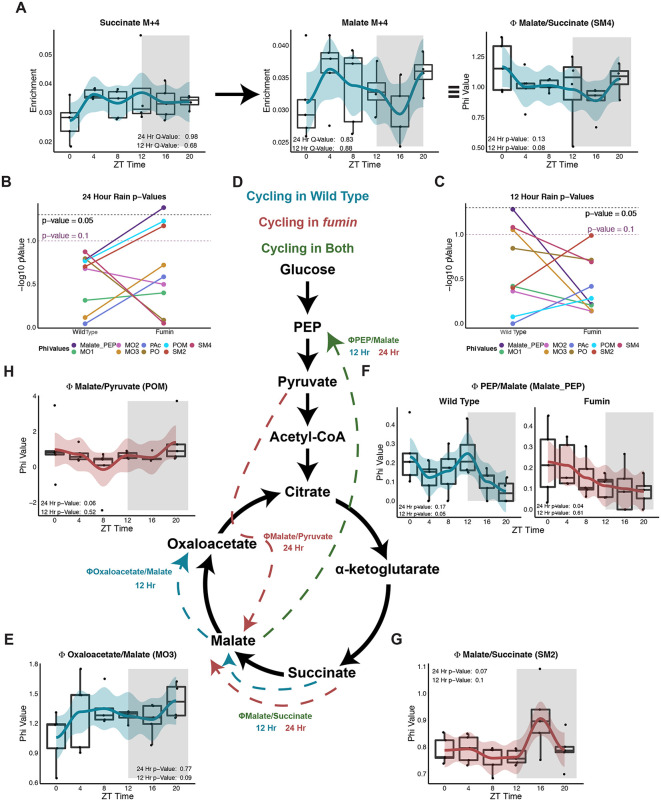
Shift from ultradian to diurnal rhythmicity is observed in product to precursor relationships in *fumin* compared to wild type. **(A)** Box plots with LOESS traces for a representative product to precursor relationship (malate M + 4/succinate M + 4) in WT (SM4, right panel), the precursor, succinate M + 4 (left), and the product, malate M + 4 (middle). N = 4 to 5 biological replicates of 15 male flies per time point. Rhythmicity was tested for 24-hour and 12-hour periods using RAIN and results with a *p*-value less than 0.1 are shown. **(B, C)** Summary of RAIN *p*-values for 24-hour (B) and 12-hour (C) periods for WT and *fmn*. Colors represent product-to-precursor relationships (phi values). A *p*-value cut-off 0.05 is shown in the black dotted line a *p*-value cut-off of 0.1 in the purple dotted line. **(D)** Overview of significant phi value relationships. **(E–H)** Box plots and LOESS traces for phi values with *p*-value less than 0.1 in WT and/or *fmn*. *n* = 4 to 5 biological replicates with 15 male flies per time point. Raw data for these figures is available as S4 Data file.

In order to understand how carbon flow was occurring through the TCA cycle, we employed a product-to-precursor relationship analysis [37]. This analysis generates ratios (phi values) using different mathematical relationships between TCA cycle precursors and products to provide further insight into how glucose is being used. For example, the SM4 ratio looks at the formation of malate M + 4 from succinate M + 4. Since malate M + 4 is only derived from succinate M + 4, the ratio provides insight into stepwise flow of oxidative carbons at this TCA cycle step relative to other pathways like anaplerotic pyruvate carboxylase. Neither of the inputs for this ratio displayed significant rhythmicity (24- or 12-hour), however, a 12-hour rhythm trending towards significance was observed in the WT ratio (Fig 7A and S4 Data). This highlights the additional value gained from the ratio analysis which was not captured at the enrichment-level alone and suggests rhythmicity between oxidative and synthetic mitochondrial metabolism.

Overall, the Malate_PEP phi value demonstrated significant 24-hour rhythmicity and two others, POM and SM2, trended towards significance in *fmn* (Fig 7B and 7D)*.* In contrast to 24-hour rhythms, 12-hour cycling trended towards significance in three WT phi values (SM4, MO3, and Malate_PEP) (Fig 7C and 7D). For SM4, a ratio of one (i.e., ZT 4) indicated the formation of malate M + 4 from succinate M + 4 was not diluted by non-oxidative carbon flows. However, a ratio greater than one (i.e., ZT 20) could indicate either reversal of the pathway or the entry of alternately labeled carbons in the formation of malate (Fig 7A middle panel). Similarly, ratios greater than one in the 12-hour rhythmic MO3 phi value suggested that either the malate was being formed from oxaloacetate or alternative routes with labeled carbons were being used to form oxaloacetate (Fig 7E). Given that decapitated fly bodies (and not single tissues) were examined and that some compartmentation is to be expected, then phis become more semi quantitative or qualitative indices of flux since true single-pool steady state assumptions may not apply. Interestingly, the Malate_PEP ratio switched from 12-hour rhythmicity in WT to 24-hour rhythmicity in *fmn* (Fig 7F) and indicates a relative shift in glycolysis versus gluconeogenesis. However, the ratio was less than one in both genotypes indicating that unlabeled carbons from alternate metabolites, such as glucose, were diluting the formation of PEP from malate. A different ratio looking at the formation of malate from succinate using the M + 2 forms, SM2, displayed 24-hour rhythmicity in *fmn* with values less than one indicating the entrance of alternate sources of unlabeled carbons (Fig 7G). *fmn* also displayed 24-hour rhythmicity in the POM ratio which looked at the formation of malate from pyruvate (Fig 7H). Here, in instances where the ratio decreased less than 1, it implied alternative sources of unlabeled carbons diluting the pathway. Overall, the product to precursor analysis highlighted a shift in key oxidative and synthetic metabolic flows from ultradian rhythmicity in WT to 24-hour rhythmicity in *fmn* pointing not only to differences in glucose utilization but also potential differences in demand.

### Feeding rhythms and a 4-hour short-term fast do not impact processing of excess glucose

**Fig 8:**
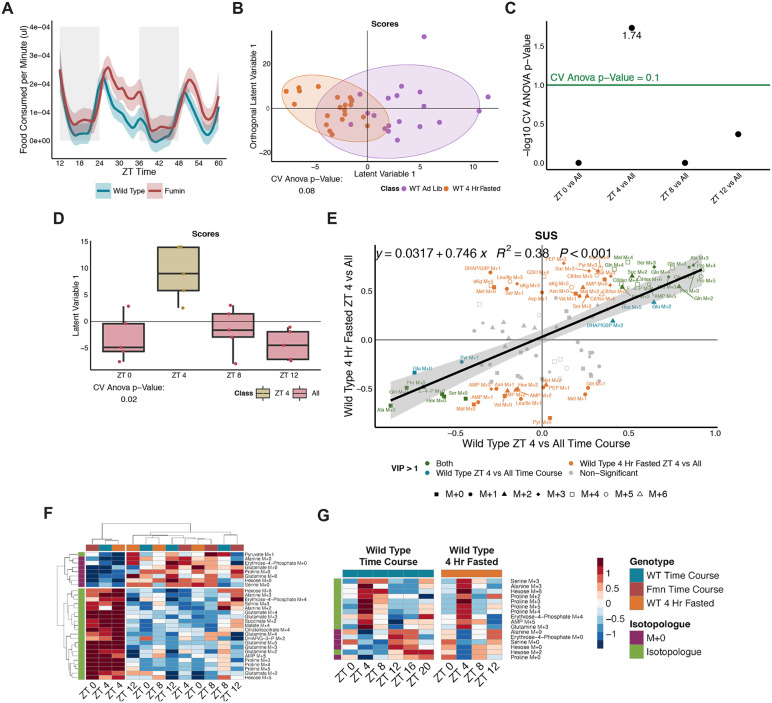
Feeding rhythms and a 4-hour short-term fast do not impact processing of excess glucose. **(A)** LOESS trace of feeding rhythms measured by volume of food consumed per minute by WT and *fmn* flies across 2 days using the ARC assay. *n* = 53 to 62 per genotype. **(B)** A two-component OPLS-DA model comparing WT *ad libitum* and short-term fasted flies. All time points collected (ZT 0, 4, 8 and 12) are included for each condition. *N* = 5 biological replicates with 18 to 24 flies per time point and group. **(C)** Negative log CV-ANOVA *p*-values for each time point tested in pairwise OPLS-DA models in the short-term fasted condition. Significance was defined by as a CV-ANOVA *p*-value less than 0.1 (shown as points above the green line). **(D)** Scores plot for the significant short-term fasted ZT 4 vs. All OPLS-DA model. Two classes were defined as ZT 4 and All (ZT 0, 8, and 12). **(E)** SUS plot comparing the loadings from the WT ZT 4 vs. All (Fig 3G) and the short-term fasted ZT 4 vs. All (S6B Fig) OPLS-DA models with a regression line and equation. Green refers to VIP compounds in both models; orange refers to VIP compounds in the short-term fasted model; blue refers to VIP compounds in the WT model (Fig 3G). Shapes represent isotopologues. **(F)** Clustered heatmap on both the x- and y-axes shown using ZT 0 to 12 time points from earlier WT and *fmn* samples along with the short-term fasted samples for VIP compounds determined from the WT ZT 4 vs. All model (Fig 3F and 3G). **(G)** Phase-ordered heatmap showing levels of 24-hour rhythmic metabolites from wild type (Fig 3B) for the short-term restricted time course. Raw data for these figures is available as S4 and S10 Data files.

Since the timing of food availability can be a zeitgeber for molecular clock rhythmicity, we asked how nutrient factors impact the time shift in biosynthesis observed between WT and *fmn*. To determine whether altered feeding rhythms between the genotypes were impacting downstream glucose biosynthesis, feeding profiles of WT and *fmn* were monitored through the Activity Recording CAFE (ARC) assay revealing similar levels of food consumption in both genotypes overall and during the light and dark periods. However, the feeding peak in *fmn* (ZT 4.5) was phase delayed in comparison to WT (ZT 3.25) (Fig 8A and S10 Data). Since the biosynthesis peak in *fmn* was observed to be phase-advanced from WT while the feeding rhythms were phase-delayed, we concluded that overall feeding was not an underlying cause of the biosynthesis shift in *fmn*.

To further establish whether the availability of food was driving biosynthesis in WT, a shortened time course from ZT 0 to ZT 12 was analyzed under *ad libitum* and a 4-hour fasted condition, where food was withheld for 4 hours prior to injection with the glucose tracer. Distinct metabolic profiles were noted for the two conditions, with different labeled isotopologue enrichments associated with each (Figs 8B and S9A). We next determined whether ZT 4 was still metabolically distinct from remaining time points under the short-term fasted condition. The ZT 4 model did emerge as significant in a pairwise discriminant model to all remaining time points (Fig 8C and 8D). Similar to the earlier observation, a majority of the labeled isotopologues were associated with ZT 4 (S9B Fig and S10 Data) pointing to increased biosynthesis from glucose. To further determine whether the presence of increased biosynthesis was arising from similar compounds and/or pathways observed previously (Fig 3G), we performed a SUS analysis using the multivariate OPLS-DA loadings from both ZT 4 versus All (WT—Fig 3G and short-term fasted—S9B Fig) models. The resulting plot showed conserved and similar changes occurring at both ZT 4 time points (highlighted in green) (Fig 8E). It is important to note that several compounds uniquely contributed to the separation of ZT 4 in the short-term fasted model, but this is to be expected as food restriction would impact metabolism and excess glucose processing. We also performed a complementary analysis using the enrichments identified from the WT ZT 4 versus All model (Fig 3G) as significantly contributing to the separation of ZT 4 from all other time points (VIP greater than 1). Using these VIP metabolites in a clustering analysis with calculated enrichments from ZT 0–12 from the WT, short-term fasted WT and *fmn* time courses, two main clusters emerged for both time and compounds (Figs 8F and S9C). On the time axis, ZT 4 from both the WT and short-term fasted condition clustered with the *fmn* ZT 0 time point while all remaining time points formed a second cluster. On the metabolite axis, labeled and unlabeled isotopologues formed the two main clusters. This clustering further highlighted the peak in labeled isotopologue enrichments at ZT 4 and ZT 0 in WT and *fmn,* respectively. Since rhythmicity analyses had also previously shown peaks in labeled isotopologues and troughs for unlabeled forms at ZT 4, we assessed the overall enrichment profiles of the short-term fasted condition using the 24-hour rhythmic metabolites (identified in Fig 3B). This further showed similar patterns across the two data sets (Fig 8G). Overall, this highlighted sustained increased downstream biosynthesis from glucose carbons and peaks in rhythmicity at ZT 4 in WT irrespective of food pointing to a potential underlying circadian driver rather than the availability of food.

## Discussion

Glucose and glucose metabolism play a central role in energy metabolism. Glucose levels are closely regulated and maintained and disruptions to glucose metabolism lead to diseases such as diabetes [38,39]. Circadian rhythms have not only been observed in glucose levels but also in processes involved in nutrient metabolism [40]. Moreover, glucose homeostasis has been shown to be impacted by deletion of a core clock component, *Bmal1*, both at a global and tissue-specific level in mice. For example, global deletion of *Bmal1* has been associated with impaired glucose tolerances and gluconeogenesis while tissue-specific deletion of *Bmal1* in the liver and intestine resulted in hypoglycemia and impaired glucose absorption, respectively [41–44]. This work demonstrated overall time-of-day differences and the presence of rhythmicity in glucose-related metabolites in blood samples from human subjects. Based on the human data, we used glucose as a tracer to begin exploring time-of-day differences in how time-dependent metabolic programming occurs in WT in comparison to the *fumin* hyperactive mutant. Although trehalose is a major carbohydrate in *Drosophila,* glucose is regulated by physiological processes and environmental cues [45]. Additionally, *Drosophila* contain homologs to mammalian insulin, *Drosophila* insulin-like peptides, insulin receptor, and insulin substrate. Because carbohydrate metabolism and signaling pathways are conserved across humans and *Drosophila, Drosophila* serves as a reasonable model to understand how glucose is processed at different times of day [46,47].

By using labeled glucose as a tracer, we have detected time-of-day differences in the fate of glucose carbons in vivo in *Drosophila.* The method used here to introduce a bolus of glucose and sample after allowing an hour for metabolism to occur may not achieve complete steady-state labeling of metabolites downstream of glucose, thus a pseudo-steady state condition is achieved which allows comparisons across times and groups to be performed. A limitation is that bolus glucose injection, while necessary to obtain robust tracer enrichment for isotopologue profiling, may introduce acute metabolic perturbations. However, we did not detect measurable behavioral disruption under this paradigm, and our primary conclusions rely on relative temporal and genotype-dependent differences. Future studies should test dose scaling and alternative tracer delivery methods to further reduce potential bolus-related artifacts. We previously reported significant 20–28-hour total metabolite pool rhythmicity in wild type under LD cycles of asparagine, glutamate, citrate/isocitrate and fructose [25]; these pools and their isotopologues were not significantly rhythmic in this study. Furthermore, cycling was not observed in metabolite pools for the isotopologues that were significant in WT. We rationalized that this difference is the result of the glucose challenge in the current study. A number of glucose-related pathways (TCA cycle, PPP, and glycolysis) were noted to use labeled glucose as indicated by the higher relative labeled isotopologue enrichments and peaks of cycling isotopologues at ZT 4. Furthermore, recent work has shown that mass action relationships primarily dictate the regulation of metabolites [48]. This supports the results observed in WT since different pathways of processing glucose are activated. However, we also observe that there is a time-of-day dependent effect where these pathways may be more active at ZT 4 allowing glucose to be cleared in a potentially more efficient manner than at other times. As glucose is sensitive to environmental cues [45], this therefore implies that underlying processes for glucose metabolism are primed at ZT 4 leading to the rhythmicity and synthesis of specific isotopologues which is not reflected in metabolite pool levels. It is important to note that the 1 M glucose load used in our tracing experiments represents a supraphysiological bolus, effectively functioning as a “metabolic stress test” or glucose tolerance challenge rather than a measure of steady-state baseline flux. This design was chosen to maximize ^13^C signal-to-noise in downstream pathways and to probe the system’s maximal capacity for glucose disposal at different times of day. Consequently, the “rush hour” we observe reflects a clock-gated window of enhanced metabolic competence (i.e., time when the system is primed to handle nutrient excess) rather than a typical rate of flux that occurs during normal feeding. While this approach robustly identifies the temporal windows of metabolic flexibility, future studies using lower-dose, continuous-labeling strategies will be valuable to map how these capacity rhythms map onto baseline nutrient handling. Although the introduction of a glucose bolus can perturb baseline rhythms and mask metabolite pool-level rhythmicity, the same pathways remain active in steady-state conditions—where their underlying rhythmicity can still be detected at the pool level. This highlights the importance of understanding how specific metabolite isotopologues are altered, which can have significant implications for understanding the interaction of the circadian clock and metabolic disease.

A key finding of this study is that the temporal regulation of glucose metabolism is fundamentally dependent on the endogenous circadian clock. While data with *fmn* mutants highlights a link between activity, sleep, and metabolic timing, it does not definitively prove clock control. Experiments using the clock-null *per⁰¹* mutant directly demonstrate the complete abolishment of rhythmic glucose flux supporting the notion that the “rush hour” is not merely a reaction to feeding patterns or light exposure but is an actively programmed event orchestrated by the molecular clock. This finding provides a critical mechanistic anchor for the metabolic phenotypes observed throughout our study. It clarifies that the altered rhythms seen in *fmn* mutants represent a modulation of a pre-existing, clock-driven metabolic program, rather than the creation of a new one. This hierarchical model in which the core clock gates metabolic pathway accessibility, helps explain how genetic or environmental disruptions that misalign physiology with the underlying circadian cycle can lead to metabolic dysfunction.

Multiple independent fly datasets support this notion of endogenous clock regulation of metabolic state, while underscoring tissue specificity. Our previous metabolomics work explicitly describes fly-body metabolite cycling alteration as a function of clock [25] disruption. Brain RNA-seq identifies several hundred circadian/diurnal cycling transcripts that are altered in *period*-null animals, indicating robust clock-dependent temporal programs in the head/brain [49]. Our previous peripheral clock studies show that a clock in the fat body drives rhythmic expression of genes involved in metabolism (with some components also influenced by exogenous cues such as feeding/light) [50]. At the organismal level, we have recently shown that integrated flow-through respirometry with metabolomics reports anticipatory metabolic/respiratory coordination in WT-LD and reactive dysregulation in *per*^*01*^ or WT-DD [51]. Because our experiments here quantify glucose-derived labeling in fly bodies following an acute bolus, they probe time-of-day differences in *flux capacity and carbon allocation* rather than steady-state abundance rhythms. As such, we treat published head/tissue omics as supportive demonstrating clock dependence and a common early-day physiological structure rather than expecting exact phase agreement across assays and tissues.

On the other hand, the number of rhythmic compounds in *fmn* was increased when compared to WT and included both isotopologues and pools. It is important to note that the presence of cycling in metabolite pools can convolute the interpretation of whether increased biosynthesis of rhythmic isotopologues is occurring specifically for the isotopologue of interest or generally of the pool. However, for 24-hour rhythmic compounds, the peak in metabolite pools aligned more closely with the peak observed in isotopologues and thus could point to the production of the isotopologues driving the rhythmicity of the pools rather than the synthesis of the metabolite through alternate pathways (other than from glucose) resulting in unlabeled forms. Further studies would be needed to definitively discriminate whether labeled carbons from metabolites other than glucose were affecting the pool sizes.

The increase in TCA cycle labeling in *fmn* may be a result of the increased activity, an increased metabolic rate [52], a switch in fuel preference, and/or decreased sleep observed in *fmn* which may necessitate an increased need for TCA intermediates and/or amino acid production. Moreover, the lack of PPP labeling in *fmn* in relation to WT overall may reflect an absence or inability to properly regulate redox oscillations as this pathway produces NADPH [53] or could simply reflect decreased glucose phosphorylation, increased gluconeogenesis and/or increased glycogenolysis. Dysfunction in the PPP can have implications for the behavior and rhythms in *fmn*. Furthermore, *fmn* was noted to process glucose differently at the LD transitions through the pathways used as well as the isotopologues generated. Similar to elevated TCA cycle labeling observed in *fmn* irrespective of time when compared to WT, glycolytic and TCA cycle related metabolites were also observed to be labeled at both ZT 0 and ZT 12 although a number of unique isotopologues to both time points were also noted. In addition to concentration being a driving factor of consumption flux [48], our results point to differences in glucose consumption pathways dependent on time-of-day and/or genotypes. Furthermore, increased energetic demands arising from hyperactivity in *fmn* may also be driving not only increased glucose utilization but also differences in glucose processing by time of day.

The results of glycogen pool and synthesis measurements suggest that altered glycogen routing/turnover may contribute to the time-resolved labeling phenotype in *fmn*, consistent with genotype-specific energetic demand, while providing less support for a model in which a ZT 4-specific deficit in glycogen storage universally explains “rush hour” labeling in control flies. Consistent with the idea that dopamine can influence glycogen metabolism, *fmn* mutants exhibit globally reduced glycogen stores across the day–night cycle, with only modest relative peaks at ZT 0 and ZT 12 compared with other time points. Thus, while dopamine-dependent control of glycogen likely tunes the overall energetic buffering capacity of the system, it cannot by itself explain the temporal reprogramming of glucose-derived flux that we uncover. Instead, our results suggest that dopamine shapes a broader, temporally fungible metabolic state in which multiple pathways rather than glycogen alone are re-aligned in time. Accordingly, we use “rush hour” as an operational term describing time-dependent tracer redistribution into measurable pools, which may reflect contributions from glycogen turnover, insect redox shuttles, and measurable allocation into lipid-associated carbon.

The striking robustness of the metabolic biosynthesis “rush hours” via isotopologue enrichments observed at ZT 4 in WT (*ad lib* or fasted) and ZT 0 in *fmn implies* that while *fmn* is slightly phase advanced in its morning glucose catabolic “program,” the overall program remains the same. We acknowledge that the 4-hour sampling interval used in our metabolomics analysis limits our ability to resolve fine-scale temporal dynamics, such as the estimated ~1.25-hour difference in peak feeding times between wild-type and *fmn* flies. Consequently, subtle metabolic phase shifts driven strictly by these minor behavioral differences may remain undetected in our dataset. However, this resolution constraint does not impact our broader conclusion that feeding time is not the primary driver of the *fmn* metabolic phenotype. Critically, the behavioral and metabolic shifts we observe are directionally opposed: while *fmn* mutants display a delay in peak feeding (occurring near ZT 6), their maximal glucose-derived biosynthetic flux is phase-advanced to the onset of light (ZT 0). This robust temporal dissociation where peak metabolic capacity actually precedes peak nutrient intake strongly suggests that the “rush hour” is orchestrated by an endogenous circadian program that has become uncoupled from behavioral rhythms, rather than being a simple downstream consequence of when the animal eats. Because *Drosophila* consume food primarily during the light period [54], one possibility for the phase advancement in catabolism is altered underlying feeding rhythms. Mistimed feeding can serve as a potent zeitgeber especially for peripheral clocks with impacts on metabolic processes [55]. We observed however, the opposite result as feeding rhythms in *fmn* were phase delayed compared to WT while the biosynthesis peak was phase advanced, feeding therefore was not the primary driver of the isotopologue rhythmicity observed. This also begins to deconvolute the influence of feeding on downstream glucose catabolism from that being driven by the presence of excess glucose and/or primed pathways. As feeding acts as a potent zeitgeber especially in peripheral clocks, misaligned feeding which can occur in shift work has been associated with altered metabolism and adverse health consequences [55,56], however; the observed phase advancement of biosynthesis also highlights the importance of understanding whether shifts in metabolism become decoupled from feeding and understanding the implications that may arise from this.

Product-to-precursor relationship analysis provides further insight into how glucose was being used in a manner that was not captured at the isotopologue level. For example, the presence of rhythmicity in a ratio was not necessarily preceded by cycling in the individual inputs. Through this analysis, we were able to observe an overall shift from 12-hour rhythmicity in WT to 24-hour rhythmicity in *fmn* which followed the activity patterns of the genotypes. Specifically, in WT, we were able to follow the flow of carbons in a stepwise manner from succinate M + 4 to malate M + 4 to oxaloacetate M + 4 through two ratios, SM4 and MO3. Both ratios looked at oxidative reactions within the TCA cycle and were around one indicating stepwise carbon flow through the reactions. However, at ZT 0 and ZT 20, the SM4 ratio was higher than one implying compartmentalization across different tissues, some error in the measurement, or incorporation of labeled bicarbonate during pyruvate carboxylation. On the other hand, *fmn* also displayed rhythmicity in oxidative reactions through the phi Value SM2. This value used M + 2 forms of malate and succinate and implied that the pathway was being diluted by alternately labeled or unlabeled carbons except at ZT 16 where the reaction was closer to a direct relationship between the product and precursor. Additionally, the POM phi value was an indicator of anaplerosis driven by pyruvate carboxylase. This phi value was close to one at ZT 0 and ZT 20 in *fmn* implying that oxaloacetate was primarily being formed through anaplerotic pathways while at the other time points, alternate sources such as malate and/or the introduction of aspartate could be contributing to the generation of oxaloacetate. Taken together, the phi value product-to-precursor relationship analysis provides additional insight into how glucose is being used in both genotypes that is not gained from typical analysis using enrichments.

While the isotope tracing portion of this study was performed in *Drosophila*, we speculate that there are important implications for understanding glucose and energy response in humans. For example, increasing evidence suggests that caloric intake biased towards earlier times have health benefits with regards to weight loss, glycemic control [57,58], and cardio-metabolic outcomes [59]. A significant challenge in the clinical application of this knowledge is overcoming lifestyle and habitual resistance changing or limiting time windows for food intake. For example, dinner is often an important social dinner component, while skipping breakfast is generally not recommended creating a difficulty in creating realistic food timing windows. Similarly, with regard to activity, overweight individuals under an acute high-fat diet have been shown to benefit from exercise with respect to cardiorespiratory fitness irrespective of timing, but only glycemic control is improved by evening exercise [60]. By understanding the downstream mechanisms at work through studies such as the current work, we may be able to eventually expand the temporal window of effectiveness of food or exercise through dietary or pharmacology intervention to more effectively employ these strategies.

Overall, we have shown the sensitivity of the metabolic fate platform to detect low-level differences in downstream glucose metabolites at different times of the day. This approach can be adapted for higher time resolution studies and/or other labeled tracers in *Drosophila*, but also in other model organisms and potentially humans due to the small amount of tracer required and high mass sensitivity of the platform. In our current study design, up to one-hour resolution can be achieved as this is the time needed for glucose to be sufficiently incorporated into downstream metabolites. More generally, this approach can be used to explore how circadian regulation of metabolism occurs in conjunction with other questions related to disease pathophysiologies, sleep, and influence of other environmental zeitgebers. Using the resulting insights, a better understanding of metabolic changes can be gained which can drive interventions in a more precise manner both in terms of targets as well as timing. While our study successfully identifies a robust, clock-gated “rush hour” of glucose metabolism, we have characterized the flux into key downstream pathways rather than building a quantitative, network-wide model. A powerful future direction will be to leverage these data to develop a time-resolved metabolic flux analysis model. Such an approach would allow for a quantitative understanding of how flux is dynamically rerouted between competing pathways—such as glycolysis versus the PPP, or anaplerotic versus cataplerotic reactions—across the circadian cycle. This represents a crucial next step toward a systems-level, mechanistic understanding of how the circadian clock orchestrates metabolism and will be essential for predicting the metabolic consequences of shift work or genetic clock disruption.

## Methods

### Ethics statement

For recruitment of human participants, Institutional Review Board of the University of Pennsylvania and the Clinical and Translational Research Center of the University of Pennsylvania granted the study protocol for subject recruitment (approval no. 807347). Written informed consent was obtained from all research subjects.

### *Drosophila* strains

*Drosophila melanogaster*, wild type (isogenic *w^1^*^*1*^*^18^* stock), *fumin* [34] and *per⁰¹* mutants [25] as characterized previously, were maintained on standard cornmeal/molasses medium at 25°C under 12:12 LD conditions.

### Geotaxis and locomotion assays

After three days under LD entrainment, 5-to-7-day-old wild type or *fumin* flies were anesthetized on ice for 15 min at ZT 0 (lights on) or 12 (lights off). Flies were either not injected (control) or injected with PBS or 1 M Glucose. For geotaxis assays [61,62], flies were placed in new vials without food for one hour after which climbing activity was measured by the time taken to climb 4 cm per fly, measured in triplicate using 5–8 flies per group. For locomotion assays [63,64], flies were placed in 5 × 65 mm glass tubes with 5% sucrose post-injections and monitored using Activity Monitoring System devices (DAMS) from Trikinetics (Waltham, MA) for a minimum of 24 hours in 25 °C LD incubators. Locomotion data was analyzed using a custom MATLAB (MathWorks, Natick, MA Versions 2013B and 2020A) script [65] with additional analysis and plotting performed in R (version 4.0.3). Comparisons for both assays were made using a two-sided *t* test using *p* < 0.05 as a threshold for significance.

### Feeding assay

An ARC assay [66] was used to record feeding rhythms of wild type and *fumin*. Male flies were entrained under 12 hours Light:Dark conditions within one day of eclosion at 21 °C. On day 6, entrained flies were used to set up the feeding assay with one fly per well containing 300 µl of 2% agar. Genotypes were loaded in alternating wells. A capillary containing a (infrared) dye (for detection of food level) and 2.5% yeast and 2.5% sucrose was placed into each well as a food source. After allowing for habituation to the setup, data was recorded for 48 hours with capillaries replaced each day before ZT 12. This assay was performed at 21 °C to minimize evaporation of the capillaries. Data was visualized in R (version 4.0.3) and any wells containing flies that had not survived, evaporation only of food and/or inconsistent food level recordings were discarded. Data was processed using a python program (Noah15.2) with additional analyses in R (version 4.0.3).

### Glucose injection time courses

Within one day after eclosion, 15–18 males were sorted into a new vial for entrainment to LD rhythms for a minimum for 3 days in appropriate incubators at 25°C. All flies were between 5–7 days old at the time of injection. One vial was placed on ice to immobilize flies 15 min prior to each injection time point. The injection solution consisted of 1 M uniformly labeled (^13^C_6_) glucose (CIL D-Glucose, 99%) and a blue dye (50× dilution of McCormick, FD&C Blue Dye No. 1) to allow for visualization during injections and normalization for variations in injection volumes during data processing. The solution was injected into the thorax using glass capillaries (9 cm × 1.14 mm diameter, Drummond Scientific, Broomall, PA) on ice. After injection, flies were moved to empty vials containing a kimwipe moistened with 1 ml of water to prevent flies from becoming dehydrated and returned to the appropriate light or dark 25 °C incubator to metabolize the glucose tracer. After one hour, flies were collected on dry ice and stored at −80 °C until extractions. Five separate days of injections were performed as biological replicates for both WT and *fumin* mutants. Any flies which died after the injections were discarded. No difference in survival rate (>95%) was noted between the genotypes.

For the shortened *ad libitum* versus 4-hour short term fasted time course, injections were modified as follows: 20–24 male wild-type flies were collected and entrained within one day of eclosion and were injected on day 7. Four hours prior to each time point, flies were transferred either to fresh food vials (*ad libitum* condition) or agar vials (4-hour fasted condition). Each group was injected with either 1 M ^13^C_6_-glucose or 1 M ^12^C_6_-glucose (Acros Organics alpha-D(+) Glucose, 99%) with blue dye (504 µM Sigma Aldrich Brilliant Blue FCF, analytical standard) dissolved in PBS.

### Metabolite extraction and LC–MS measurements

Fly heads and bodies were separated prior to metabolite extraction, using an adaptation of the Bligh-dyer extraction [35,67]. Briefly, to each fly body sample, a total of 600 µL of cold 2:1 methanol:chloroform was added and homogenized in a bead-based tissue homogenizer at 25 Hz for 4 min (TissuLyser II, Qiagen, Hilden, Germany). 200 µL each of water and chloroform were then added, followed by centrifugation at 18787*g* for 7–10 min at 4 °C. 350–400 µL of the upper (aqueous) layer was collected and dried under vacuum until dry or overnight. Samples were resuspended at 4 µl/fly body for negative mode and diluted to 5.33 µl/fly body for positive mode using 50:50 water:acetonitrile. Each sample was analyzed separately in ESI positive and negative modes. Transitions included in each method are shown in Table A in S4 and S10 Data files. Chromatographic separations utilized an ACQUITY UPLC BEH Amide column (2.1 × 150 mm, 1.7 µm) on a Waters ACQUITY H-Class UPLC coupled to a triple quadrupole Waters Xevo TQ-S Micro MS (Milford, MA). LC conditions for the positive-ionizing method were performed as described previously [68] with a modified gradient from 100% to 20.6% B over 15 min at 0.35 mL/min, followed by a wash of 100% A for 5 min. Mobile phase B was changed from 0% to 100% from 20 to 22 min and held for column equilibration until 30 min. For the negative-ionizing method, solvents consisted of 95:5 water:acetonitrile with 20 mM ammonium bicarbonate, pH 9 (mobile phase A) and 90:10 water:acetonitrile with 20 mM ammonium bicarbonate, pH 9 (mobile phase A). The gradient was changed from 100% to 20.6% B over 15 min at 0.4 mL/min, followed by a wash of 100% A for 5 min. Mobile phase B was changed from 0% to 100% from 20 to 22 min and held for column equilibration until 30 min. Ion counts were acquired through multiple reaction monitoring (MRM).

For the feeding time course samples, the LC–MS analyses was performed as described above with the addition of 5 µM ammonium phosphate to both mobile phases in both ionization modes.

### Data processing and normalization

Chromatograms were processed using TargetLynx under MassLynx version 4.1 or using El-MAVEN (version 0.12.1 beta) and exported as ion counts for further processing in R (version 4.0.3). Column pressure deviations were monitored to identify poor sample injections which were excluded from further analysis. MRMs which were either not detected or overlapped with other chromatographic peaks and thus prevented unequivocal peak integration were set to zero. For each sample, analytical duplicates were injected in a randomized order, along with QC samples every 4–10 injections, which was comprised of a pool of all samples.

In order to perform an instrumental drift correction, consecutive QCs were averaged. Next, ion counts were used to calculate metabolite pools and were also used to calculate a ratio of each transition acquired to the calculated pool. A LOESS correction was performed on the metabolite pools to minimize the impact of differences in detection of isotopologues across samples [69]. The corrected pools were multiplied by the ratios calculated earlier to regenerate transition-level data corrected for instrumental drift. A validated FCF transition from the negative mode data was used to normalize for variation in glucose injection volumes for the wild type and *fumin* time courses. Analytical replicates were then averaged and used to perform a NA correction using the Polly Phi LC–MS/MS application [70,71]. Transitions were summed to obtain isotopologue-level data which was then used to calculate enrichments of each isotopologue on a metabolite level. Enrichments were used for multivariate and rhythmicity analyses. The NA-corrected data was also used to calculate phi values (product/precursor relationships) using the Polly Phi LC–MS/MS application. The resulting ratios were used for rhythmicity analyses.

The *ad libitum* versus short-term fasted time course data acquired validated FCF transitions in both ESI negative and positive modes and were used to normalize the data within each mode. After averaging the analytical replicates, a background correction was performed using the Polly Phi LC–MS/MS application using the ^12^C injected samples as background samples for the ^13^C injected samples. The background-corrected data was then corrected for NA.

### Multivariate and rhythmicity analyses

Principal component analyses through SIMCA (Umetrics, version 17) were periodically checked throughout the data processing steps to ensure data quality was being maintained. Enrichments were used for further OPLS-DA models in SIMCA. OPLS-DA models were generated to test for time-dependent within each genotype and/or group by defining each time point as a class with remaining time points as a second class. Genotype/Group level differences were also tested overall as well as for each time point. For each model, the number of components was trimmed to obtain a model with the lowest CV-ANOVA *p*-value to avoid overfitting. A CV-ANOVA *p*-value cutoff of 0.1 was used to determine significant models for further analysis. SUS analyses [72] were also performed in SIMCA using significant OPLS-DA models. Each metabolite enrichment was treated as an independent variable in multivariate models for computational simplicity.

In addition to multivariate analyses, univariate analyses were also performed. A two-way ANOVA using genotype and time was performed followed by a post-hoc Tukey HSD test for any significant interaction compounds. *P*-values were adjusted using a BH correction. The adjusted ANOVA genotype *p*-values along with calculated fold changes of *fumin* to wild type were used in a volcano plot.

To identify metabolites with 24-h rhythmic patterns, we applied two complementary rhythm-detection methods (JTK_CYCLE and RAIN), which test whether a time series follows a repeating daily pattern while accounting for the sampling interval. Using both approaches provides a robustness check, as each method has different sensitivities to waveform shape and noise. Using the enrichment-level data as 5 biological replicates from ZT 0–20, RAIN [73] and JTK [74,75] algorithms were used to assess diurnal (24- and 20–28-hour periods) and ultradian (12-hour) rhythmicity through the R Rain and MetaCycle packages, respectively. Results from RAIN using a *q*-value cutoff of 0.2 were used for further analyses. For the product to precursor relationships (phi values), RAIN *p*-values less than 0.1 were used to determine significance. For feeding data, rhythms were tested using food consumption combined into 10-minute bins through JTK.

### FCF and glucose quantitation

Representative sample of wild-type flies were injected with a glucose and blue dye solution, collected, and extracted as described earlier. Uninjected flies were collected and extracted to use as a background matrix for the calibration curve. To ensure a similar background, the upper fractions from uninjected samples were pooled and then aliquoted before drying. Injected samples were resuspended in diluent while uninjected samples were resuspended in appropriate calibration solutions containing varying FCF concentrations in the diluent. Integration was performed in El-Maven as described with data processing in R (version 4.0.3) and Prism (version 9).

### Human sample analysis

Plasma samples from 14 patients (8 males and 6 females; 12 White; average standard deviation age 29.14 9.37) collected as described in [76] were used. These samples were collected every four hours from an in-patient protocol and the first 24 h of samples analyzed here. The consent of the Institutional Review Board of the University of Pennsylvania and the Clinical and Translational Research Center of the University of Pennsylvania was granted for this study and informed consent obtained from all research subjects. 150 µL of plasma was used as a starting material and extracted using the method described in metabolite extraction and LC–MS measurements section with the following differences: To each sample 900 µL of 2:1 methanol:chloroform was added followed by 300 µL of chloroform and water. Samples were vortexed and centrifuged and 200 µL of the upper fraction was collected for LC–MS analyses and dried for 6 hours under vacuum. Paired samples were acquired using an ion-switching method using transitions, solvents, instrumentation, LC and MS parameters described in [35]. For sample resuspension, dried upper fractions were resuspending using 200 µL of 15:85 milliQ water:acetonitrile. Acquired data was integrated using El-MAVEN (version 0.12.1 beta) as described in the data processing and normalization section. Exported ion counts were corrected for instrument drift using a linear correction followed by median fold change normalization. The data was combined for all subjects into a single dataset. To normalize for interindividual differences in concentration, the ratio of each metabolite to the average for that metabolite was taken on an individual level. The resulting ratios were used for multivariate and rhythmicity analyses as described in the multivariate and rhythmicity analysis section.

### Pathway analysis

Significant pathways as identified by VIP values were used in MetaboAnalyst 5.0 for a pathway analysis [77]. Metabolites were uploaded using HMDB identifiers and processed using a hypergeometric enrichment method, relative-betweenness centrality for topology analysis using the Homo sapiens (KEGG) pathway library. The resulting data was imported into R (Version 4.0.3) and further analyzed. Significant pathways were defined using an FDR less than 0.05. To be included in further analyses, an impact value greater than zero was required for at least one time comparison.

### Total glycogen measurement

Glycogen measurements were done as previously [78] documented. Fly bodies of 5- to 7-day-old adult male flies were collected on dry ice prior to homogenization. The decapitated fly bodies (*N* = 3 groups/ five flies each group/genotype/time point) homogenized in 200 µl of 0.1 M NaOH. The homogenate was then centrifuged at 13,300 rpm for 10 min at 4 °C. The supernatant was extracted from each replicate separately and 20 µl of the lysate was treated with 5 mg/ml Amyloglucosidase (Sigma-Aldrich, CAS-No-9032-08-0) in 0.2 M acetate, pH 4.8. Amyloglucosidase enzyme catabolizes glycogen to yield free glucose molecules. Simultaneously, another 20 ul aliquot of the lysate was treated with 0.2 M acetate, pH 4.8 alone (untreated lysate). Both reactions incubated for 2 h at 37 °C on the heating blocks. Subsequently, Amplex Red Glucose/Glucose Oxidase Assay kit (Invitrogen-A22189) was used to measure the free glucose content in each reaction in triplicate. The protein concentration of these reactions was then measured using the Pierce^TM^ BCA Protein Assay Kit (Thermo Scientific, Rockford, IL) for normalization. Finally, the glycogen content of each sample was calculated by subtracting the free glucose concentration of the untreated lysate from the free glucose concentration of the lysate that was treated with amyloglucosidase.

### Glycogen enrichment analysis

Lysates post Amyloglucosidase reaction, as mentioned in previous section, was subjected to Bligh-Dyer extraction. Polar fraction of the extract was evaporated, reconstituted in 98:2 methanol/water, and 2.5 μl spotted on PTFE-coated glass slides for further analysis in DESI-MS.

Glucose isotopomer intensities were obtained from DESI-MS analysis of fly body extracts and exported as peak areas for the [M + ³⁵Cl]^−^ adduct at *m*/*z* 215.0328–221.0529 (M + 0 to M + 6). For each sample, the total isotopomer pool was calculated as the sum of the M + 0–M + 6 signals, and fractional enrichment was defined as the M + 6 fraction of this pool (M6/pool = M + 6 ÷ ΣM + 0–M + 6). Flies were sampled every 4 h from ZT 0 to ZT 20 with three biological replicates per group (*fmn* and wild type) at each time point (40 male fly bodies each replicate/genotype/time point). To visualize multi-day dynamics, these replicates were ordered by sample identifier and mapped onto three consecutive “virtual” days (replicate 1: ZT 0–20 hours, replicate 2: ZT 24–44 hours, replicate 3: ZT 48–68 hours), yielding a continuous 72 hours pseudo-time series for each group.

### Proline supplementation

Flies were reared under standard conditions (25 °C, 12 hours:12 hours light–dark cycle) on conventional cornmeal–molasses food. 2–4-day-old adult males were used for the experiments. For proline supplementation, L-proline (Sigma) was dissolved in distilled water to prepare a 1 M stock solution. The stock was added to melted, cooled (~50 °C) fly food to a final concentration of 30 mM and 15 mM, mixed thoroughly, and poured into standard vials.

For each genotype, flies were transferred to vials containing 30 or 15 mM proline-supplemented food (“proline media”) and allowed to feed *ad libitum* for 5 consecutive days. Multiple vials contained 20 flies each, and flies were transferred to fresh 30 and 15 mM proline food every 2–3 days as needed to maintain food quality. Only flies that survived on 30 and 15 mM proline through the pre-feeding period were used for subsequent glucose injection experiments and controlled condition. On day 6 (Day 0 on graphs) of proline pre-feeding, surviving flies from each genotype were randomly allocated to experimental and control groups: ^12^C **Glucose-injected groups (Proline + Glucose):** For each genotype, three independent biological replicates were set up, each consisting of 20–25 flies (*n* = 3 vials/genotype). Flies were kept briefly on ice and injected with a ^12^C-glucose solution as described in the “Glucose injection and metabolic labeling” section. Following injection, flies were immediately returned to fresh 30 and 15 mM proline food and maintained under standard conditions. **Non-injected control groups (Proline only):** In parallel, for each genotype, three independent biological replicates of 20–25 flies each (*n* = 3 vials/genotype) were handled identically (including ice exposure, if matched to injected groups), but were not injected. These flies were also maintained on 30 and 15 mM proline food for the duration of the assay. After the day 6 intervention (glucose injection or control handling), survival was monitored daily for at least 3 days. Dead flies were counted and removed at the same circadian time each day, and the number of surviving flies per vial was recorded. Flies were maintained on 30 and 15 mM proline food throughout this period, with food changed if necessary to prevent desiccation or mold. For each genotype and condition, survival was summarized as the mean ± SEM across the three biological replicates. Genotype- and treatment-dependent effects on survival at each time point (e.g., days 2 and 3 post-injection) were assessed using appropriate statistical tests (ANOVA Sidak’s multiple comparison test).

## Supporting information

S1 Fig Pairwise OPLS-DA models for each time point and 20–28-hour rhythmicity support time-of-day variation in metabolite levels. **(A)** Overview of study design showing clock times and corresponding ZT times for blood collections adapted from [76]. **(B–F)** Scores plots (left panel) and corresponding loadings plots (right panel) for significant pairwise OPLS-DA models. Colors in scores plots represent classes and in loadings plot represent classes of metabolites. Size of points in loadings plot represents significance as defined through VIP values. Models are (B) ZT 0 versus All, (C) ZT 8 versus All, (D) ZT 12 versus All, (E) ZT 16 versus All, and (F) ZT 20 versus All. Two classes were defined for each model as the time point being tested and all remaining time points. **(G)** Overview of the number of significant 20–28-hour rhythmic metabolites observed at RAIN (dark) or JTK (light) *q*-value cut-offs of 0.1, 0.2, 0.3, and 0.4. **(H)** Phase-ordered heatmap of significantly cycling metabolites with 20–28-hour periods as tested by RAIN with a *q*-value less than 0.2. **(I)** Distribution of RAIN phases for significant 20–28-hour metabolites. Colors represent classes of metabolites. Raw data for these figures is available as S1 Data file.
(PDF)

S2 Fig Overview of 20–28-hour and 12-hour rhythmicity in wild type. **(A, D)** Overview of the number of significant 12-hour (a) and 20–28-hour (d) rhythmic compounds observed at RAIN *q*-values of 0.1, 0.2, 0.3, and 0.4. **(B, E)** Phase-ordered heatmaps of significantly cycling compounds with 12-hour (B) and 20–28-hour (E) periods in wild type as tested by RAIN with a *q*-value less than 0.2. **(C, F)** Distribution of RAIN phases for significant 12-hour (C) and 20–28-hour (F) compounds grouped by metabolite. Shapes and colors refer to isotopologues or pools. Raw data for these figures is available as S4 Data file.
(PDF)

S3 Fig Time-of-day-dependent incorporation of glucose-derived carbon into lipid-associated NMR resonances and altered ^13^C-hexose labeling dynamics in *per*^*01*^. **(A)** Representative ^1^H–^13^C HSQC spectrum from flies injected with [U-^13^C]-glucose, showing the set of manually defined regions of interest (ROIs) used to quantify label incorporation into major aliphatic resonances. ROIs are outlined in green. ROI2, highlighted here, corresponds to a cluster of cross-peaks in the lipid-associated aliphatic region (primarily CH₃/CH₂ environments). **(B)** Heatmap of temporal changes in the labeled–unlabeled intensity difference across all HSQC ROIs over the circadian cycle. Warmer colors indicate increased ^13^C incorporation relative to unlabeled controls. ROI2 exhibits a delayed peak in ^13^C enrichment relative to small-molecule metabolites, consistent with precursor to lipid allocation following the early-day metabolic “rush hour.” **(C)** Fraction of labeled hexose (1 − M⁰/total isotopologue pool) across circadian time for WT and *per* mutants under LD following [U-^13^C]-glucose injection. Boxplots show individual biological replicates; lines represent LOESS fits ± 95% CI. WT flies exhibit a time-structured labeling profile with an early-day rise in fractional labeling, whereas *per* mutants show attenuated temporal structure consistent with loss of circadian regulation of glucose handling and downstream carbon allocation. Raw data for these figures is available as S5 (A and B) and S6 (C) Data files.
(PDF)

S4 Fig Genotype differences at ZT 4 display common and unique changes in labeled metabolite forms. **(A)** Heatmap showing pearson correlation of select isotopologues with glutathione M + 5 for both wild type and *fumin*. **(B)** Negative log CV-ANOVA *p*-values for each time point tested in pairwise OPLS-DA models between genotypes. Significance was defined as a CV-ANOVA *p*-value less than 0.1 (shown as points above the green line). **(C)** Scores plot of the significant ZT 4 wild type versus *fumin* OPLS-DA model. Classes were defined as all ZT 4 samples for WT as one class and for *fumin* as a second class. **(D)** Loadings plot of the corresponding ZT 4 wild type versus *fumin* OPLS-DA model. Colors represent different isotopologues while shading representing VIP significance (dark signifies a VIP value greater than 1 while lighter shades are used for a VIP less than 1). Abbreviations are defined in Fig 3G. Raw data for these figures is available as S4 Data file.
(PDF)

S5 Fig Increased biosynthesis downstream of glucose is observed during the light period in *fumin.* **(A)** Scores plot of the significant *fumin* Light (ZT 4 and ZT 8) versus Dark (ZT 16 and ZT 20) OPLS-DA model. **(B)** Corresponding loadings plot for the *fumin* Light versus Dark scores (A) plot. Colors represent different isotopologues and shading represents VIP significance. Abbreviations are defined in Fig 3G. **(C)** SUS plot comparing the loadings plot from the Wild Type ZT 4 versus All (Fig 3G) and *fumin* ZT 12 versus All (Fig 5E) OPLS-DA models with a regression line and equation. Green refers to VIP compounds in both models; blue refers to VIP compounds in the wild type model; red refers to VIP compounds in the *fumin* model. Shapes refer to isotopologues. Abbreviations are defined in Fig 3G. Raw data for these figures is available as S4 Data file.
(PDF)

S6 Fig Time-dependent lethality of *fumin* flies on proline with and without ^12^C-glucose injection. **(A–D)** Survival of wild-type and *fumin* flies fed 30 mM L-proline at ZT 4 (A, B) or ZT 16 (C, D) under control conditions (no injection) or following ^12^C-glucose injection. At ZT 4, proline feeding resulted in selective lethality of *fumin* flies under control conditions (A) but not after glucose injection (B). At ZT 16, *fumin*-specific lethality emerged only in the post-injection condition (D). **(E–H)** Survival of wild-type and *fumin* flies fed 15 mM L-proline at ZT 4 (E, F) or ZT 16 (G, H). Data represent *N* = 3 biological replicates (20–25 flies each replicate) per genotype per condition. Statistical analysis was performed using ANOVA Sidak’s multiple comparison test for obtaining *P*-values: *p < 0.05, **p < 0.005, and ***p < 0.0005. For all graphs, error bars = SEM and NS is not significant. Raw data for these figures is available as S7 Data file.
(PDF)

S7 Fig Dopamine transporter mutant *fumin* show globally reduced glycogen that does not explain the shifted metabolic “rush hour”. **(A)** Ratio of ^13^C-labeled hexose (M + 6) to total glycogen-derived glucose following enzymatic hydrolysis of whole-body glycogen in LD. Flies were injected with [U-^13^C]-glucose at each time point (*n* = 3/time point, shown as separate days), and glycogen-derived label was quantified by DESI–MS after hydrolysis. Shaded vertical bars denote subjective nighttime. **(B)** Quantification of glycogen levels in wild-type and *fumin* flies over a 24 hour light–dark cycle (ZT 0, 4, 8, 12, 16, 20). Glycogen levels were measured in 5-to 7-day—old males for both the genotypes. The resultant values were then normalized to protein content. Fly heads were removed prior to homogenization. *N* = 3 replicates. Each sample contained an independent group of 4 fly bodies. Within the mutant, glycogen showed a modest bimodal pattern, with ZT 0 and ZT 12 representing relative higher levels compared with other ZTs. Notably, these relative glycogen peaks in *fumin* did not coincide with the time window of maximal post-glucose-challenge flux, indicating that the shifted “rush hour” cannot be simply attributed to a change in glycogen synthesis or storage timing. Statistical analysis was performed using ANOVA Sidak’s multiple comparison test for obtaining *P*-values: *p < 0.05, **p < 0.005, and ***p < 0.0005 for **B**. For **C** and **D**, statistical analysis was performed using ANOVA Tukey’s multiple comparison test for obtaining *P*-values: *p < 0.05, **p < 0.005, and ***p < 0.0005. For all graphs, error bars = SEM and NS is not significant. Raw data for these figures is available as S8 (A) and S9 (B–D) Data files.
(PDF)

S8 Fig 20–28-hour rhythmicity in *fumin* complements/supports 24-hour rhythmicity. **(A)** Overview of the number of significant 20–28-hour rhythmic compounds observed at RAIN (dark) or JTK (light) *q*-value cut-offs of 0.1, 0.2, 0.3, and 0.4. **(B)** Phase-ordered heatmaps of significantly cycling compounds with periods of 20–28-hour in *fumin* at a RAIN *q*-value cutoff of 0.2. Enrichments for wild type are shown for comparison. **(C)** Distribution of RAIN phases for significant 20–28-hour compounds in *fumin* grouped by metabolite. Shapes and colors refer to isotopologues or pools. **(D)** Comparison of 20–28-hour significant cycling compounds in wild type and *fumin* as defined by a RAIN *q*-value less than 0.2. *q*-value cutoffs of 0.2 (black dotted line) and 0.4 (purple dotted line) are shown. Color refers to significant rhythmicity in one or both genotypes while shapes refer to isotopologues. Abbreviations are defined in Fig 3G. Raw data for these figures is available as S4 Data file.
(PDF)

S9 Fig Changes in isotopologue enrichments in response to a 4-hour short-term fast. **(A)** Corresponding loadings plot for the *ad libitum* versus short-term fasted condition. Isotopologues are represented by shapes, metabolites by color and size of the shapes represents significance as defined through VIP values. Abbreviations as defined in Fig 3G; Isocitrate (Iso); Fructose-1,6-Phosphate (F-1,6-P); Fructose (Fruc); Fumarate (Fum); Glucose (Gluc); Hexose-6-Phosphate (H-6-P); Phosphoglycerates (PG); Ribulose-5-Phosphate (R-5-P). **(B)** Corresponding loadings plot for the short-term fasted ZT 4 versus All OPLS-DA model. Colors represent isotopologues with shading representing VIP significance (dark signifies a VIP value greater than 1 while lighter shades are used for a VIP less than 1). Abbreviations as defined in Fig 3G and 3A. **(C)** Clustered heatmap on both the x and y-axes shown using ZT 0–12 time points from the earlier Wild Type samples along with the short-term fasted samples for VIP compounds determined from the Wild Type ZT 4 versus All model (Fig 3F and 3G). Raw data for these figures is available as S4 and S10 Data files.
(PDF)

S1 Data **Table A.** Human data—All sets and transitions combined (no zeroes). **Table B.** Sample information.
(XLSX)

S2 Data **Table A.** Geotaxis results for wild type. **Table B.** Geotaxis results for *fumin.*
(XLSX)

S3 Data **Table A.** Locomotion data for wild type (ZT 0). **Table B.** Locomotion data for wild type (ZT 12). **Table C.** Locomotion data for *fumin* (ZT 0). **Table D.** Locomotion data for *fumin* (ZT 12). **Table E.** Male activity data for wild type and *fumin.*
(XLSX)

S4 Data **Table A.** Transitions for wild type and *fumin* time courses. **Table B.** Positive mode raw data for wild type and *fumin* time courses. **Table C.** Negative mode raw data for wild type and *fumin* time courses. **Table D.** Enrichment and pool values for wild type and *fumin* time courses.
(XLSX)

S5 Data ROI2 points from lipid synthesis. (XLSX)

S6 Data LCMS Hexose isotopologues. (XLSX)

S7 Data Time-dependent lethality raw data of wild type and *fumin* flies on proline. (XLSX)

S8 Data Results of Glycogen-associated labeling. (XLSX)

S9 Data Total glycogen assay raw data for wild type and *fumin.* (XLSX)

S10 Data Table A. Transitions for wild-type *ad libitum* and 4-hour fasted time courses. Table B. Positive mode raw data for wild type *ad libitum* and 4-hour fasted time courses. Table C. Negative mode raw data for wild type *ad libitum* and 4-hour fasted time courses. Table D. Enrichment and pool values for wild-type *ad libitum* and 4-hour fasted time courses. Table E. Feeding raw data.
(XLSX)

## Abbreviations

ARC: An Activity Recording CAFE
BH: Benjamini–Hochberg
MRM: multiple reaction monitoring
NA: natural abundance
OPLS-DA: orthogonal partial least squares-discriminant analysis
PPP: pentose phosphate pathway
SUS: shared and unique structures
QC: quality control
ZT: zeitgeber time
