# Supplementary figures and images for: Glucose is dynamically regulated by time of day in humans and Drosophila
Source: PLoS Biol. 2026 Apr 16;24(4):e3003717. doi: 10.1371/journal.pbio.3003717 (PMC13086310; doi:10.1371/journal.pbio.3003717)

A

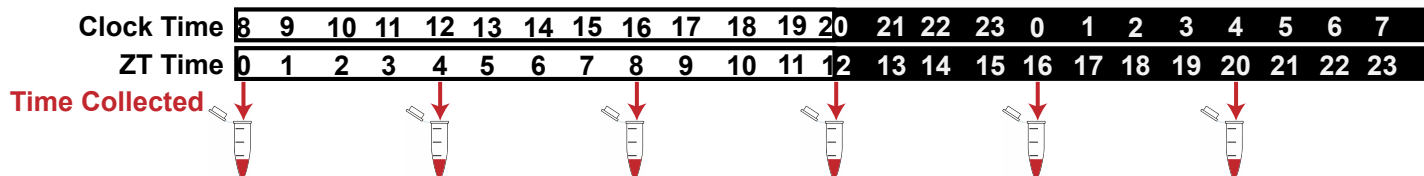

B

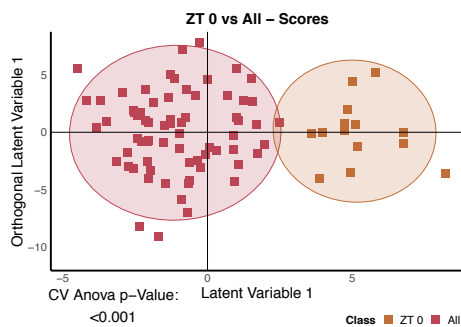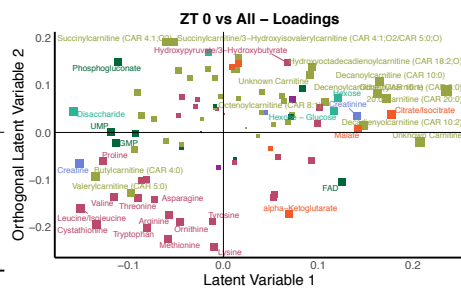

G

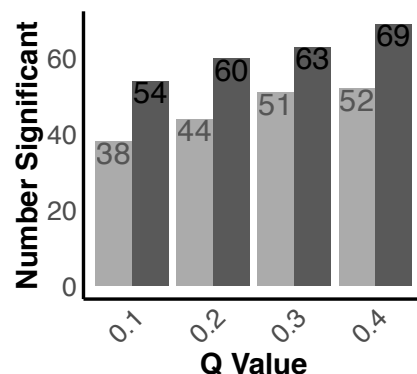

C

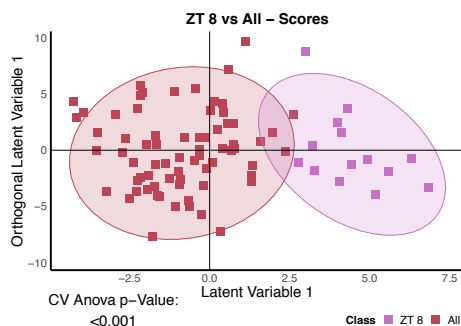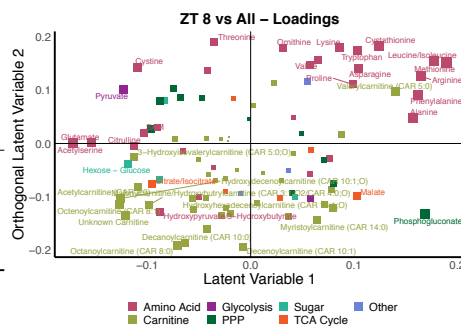

D

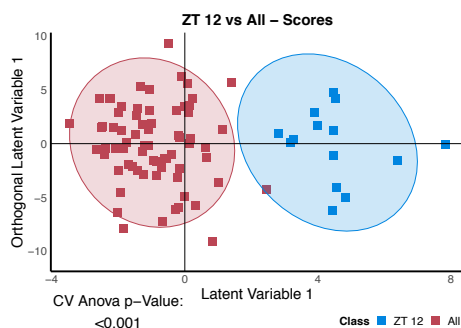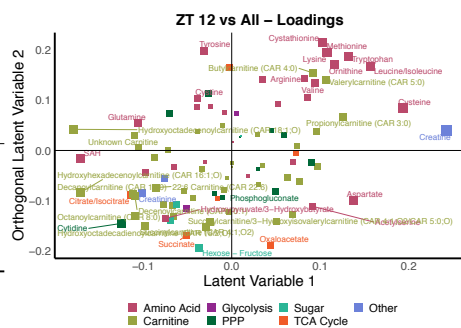

E

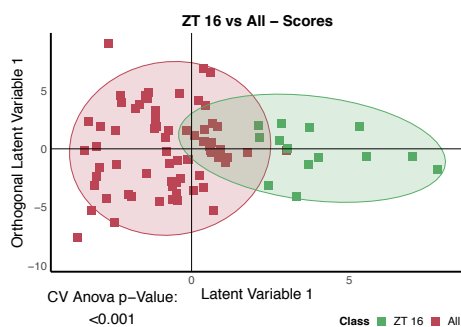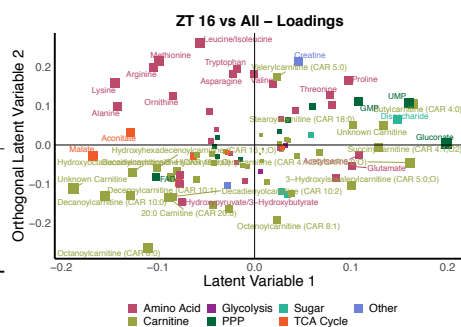

F

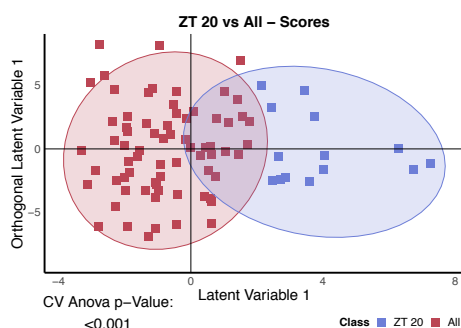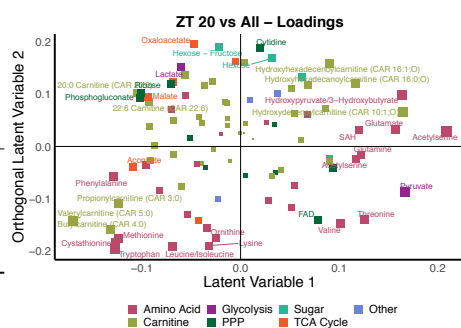

H

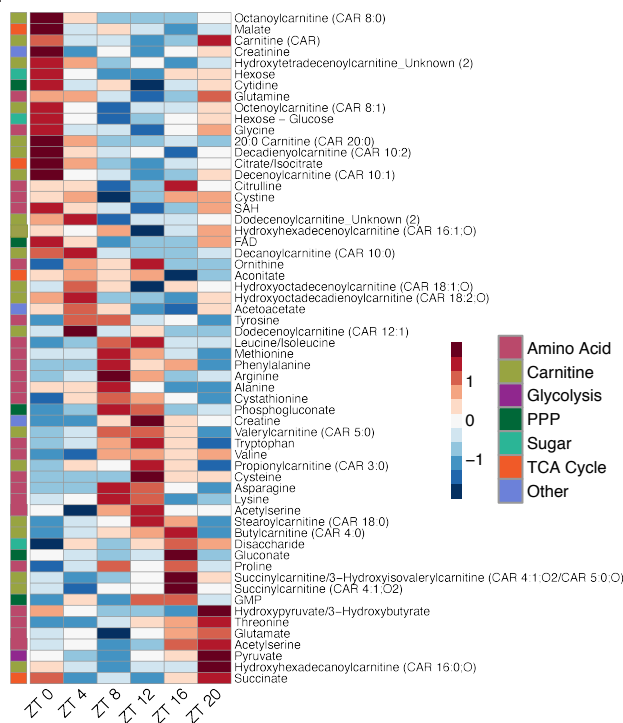

I

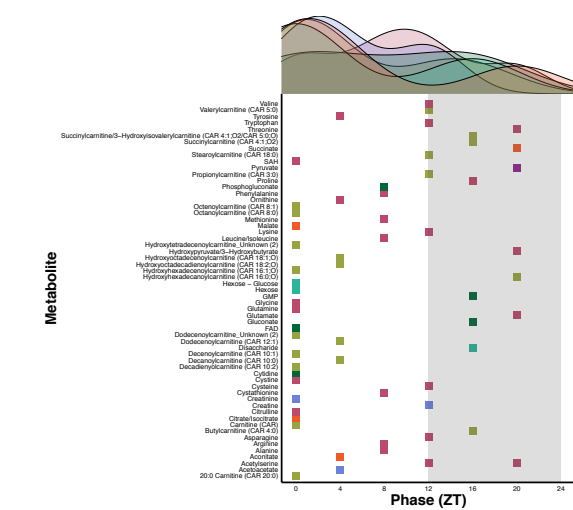

Supplement: S1 Fig — (A) Overview of study design showing clock times and corresponding ZT times for blood collections adapted from [76]. (B–F) Scores plots (left panel) and corresponding loadings plots (right panel) for significant pairwise OPLS-DA models. Colors in scores plots represent classes and in loadings plot represent classes of metabolites. Size of points in loadings plot represents significance as defined through VIP values. Models are (B) ZT 0 versus All, (C) ZT 8 versus All, (D) ZT 12 versus All, (E) ZT 16 versus All, and (F) ZT 20 versus All. Two classes were defined for each model as the time point being tested and all remaining time points. (G) Overview of the number of significant 20–28-hour rhythmic metabolites observed at RAIN (dark) or JTK (light) q-value cut-offs of 0.1, 0.2, 0.3, and 0.4. (H) Phase-ordered heatmap of significantly cycling metabolites with 20–28-hour periods as tested by RAIN with a q-value less than 0.2. (I) Distribution of RAIN phases for significant 20–28-hour metabolites. Colors represent classes of metabolites. Raw data for these figures is available as S1 Data file. (PDF) [file pbio.3003717.s001.pdf]

## 20 - 28 Hr Rhythmicity

A

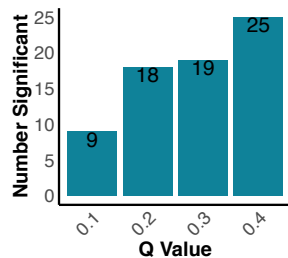

B

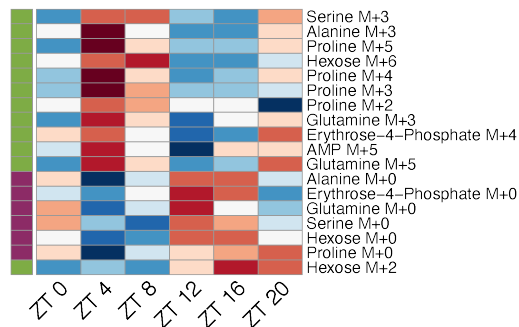

C

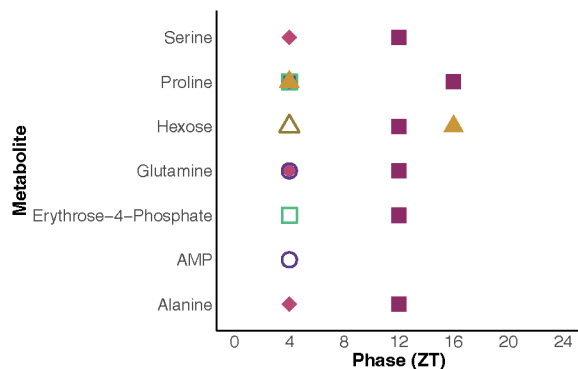

Isotopologue  
M+0  
Isotopologue

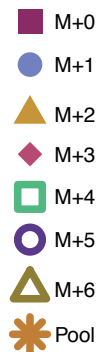

## 12 Hr Rhythmicity

D

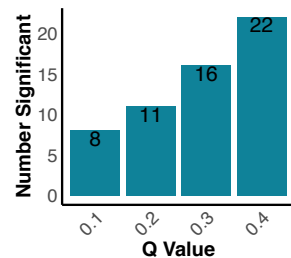

E

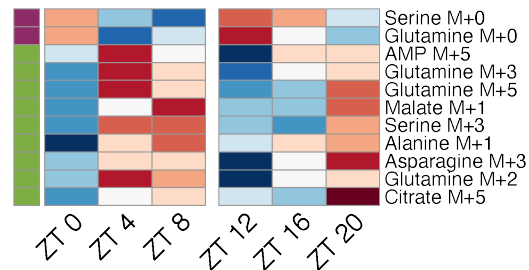

F

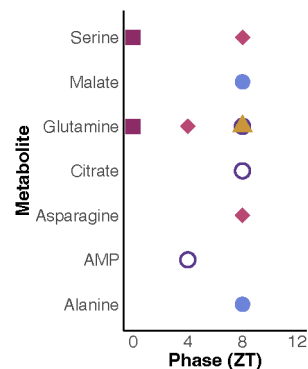

Supplement: S2 Fig — (A, D) Overview of the number of significant 12-hour (a) and 20–28-hour (d) rhythmic compounds observed at RAIN q-values of 0.1, 0.2, 0.3, and 0.4. (B, E) Phase-ordered heatmaps of significantly cycling compounds with 12-hour (B) and 20–28-hour (E) periods in wild type as tested by RAIN with a q-value less than 0.2. (C, F) Distribution of RAIN phases for significant 12-hour (C) and 20–28-hour (F) compounds grouped by metabolite. Shapes and colors refer to isotopologues or pools. Raw data for these figures is available as S4 Data file. (PDF) [file pbio.3003717.s002.pdf]

**A**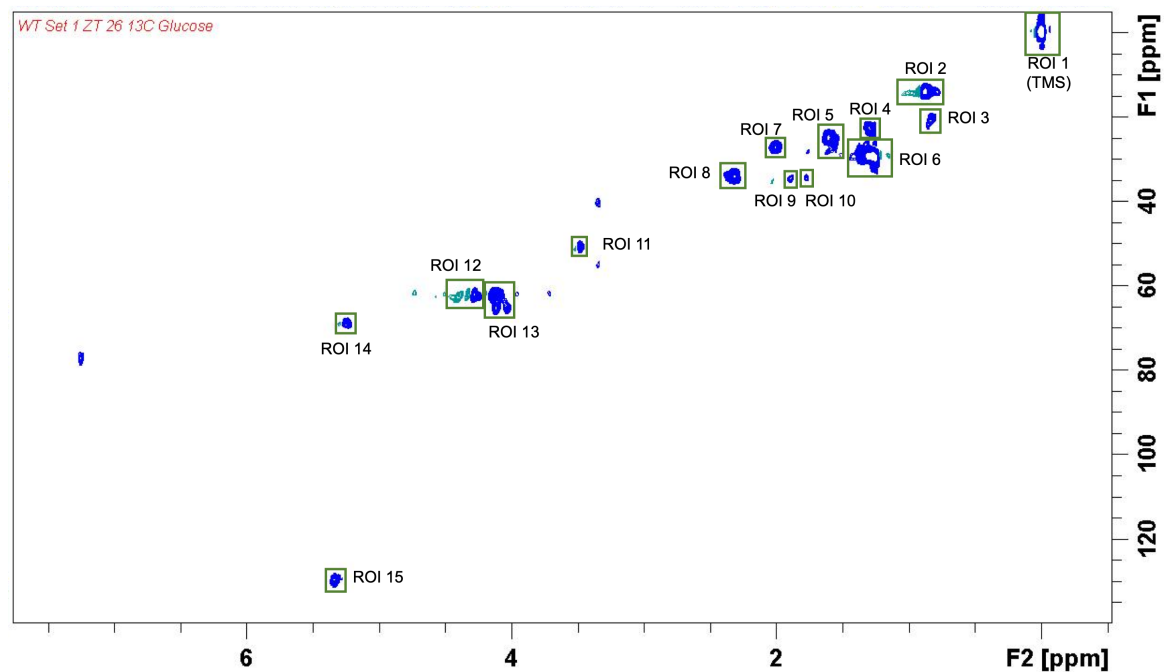**B**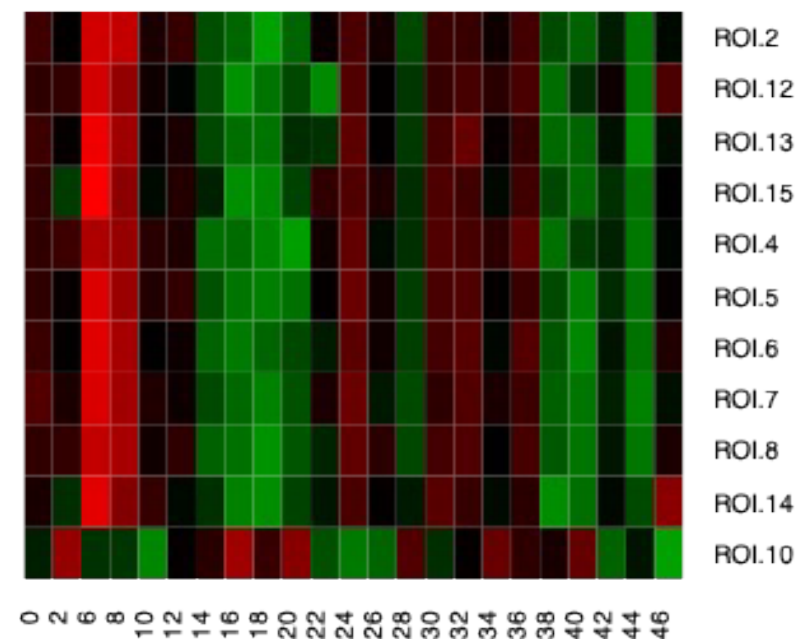**C**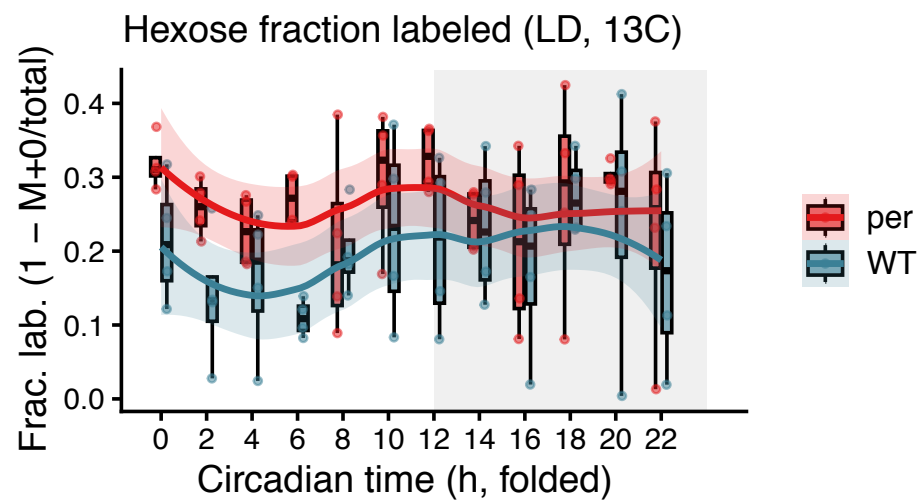

Supplement: S3 Fig — (A) Representative 1H–13C HSQC spectrum from flies injected with [U-13C]-glucose, showing the set of manually defined regions of interest (ROIs) used to quantify label incorporation into major aliphatic resonances. ROIs are outlined in green. ROI2, highlighted here, corresponds to a cluster of cross-peaks in the lipid-associated aliphatic region (primarily CH₃/CH₂ environments). (B) Heatmap of temporal changes in the labeled–unlabeled intensity difference across all HSQC ROIs over the circadian cycle. Warmer colors indicate increased 13C incorporation relative to unlabeled controls. ROI2 exhibits a delayed peak in 13C enrichment relative to small-molecule metabolites, consistent with precursor to lipid allocation following the early-day metabolic “rush hour.” (C) Fraction of labeled hexose (1 − M⁰/total isotopologue pool) across circadian time for WT and per mutants under LD following [U-13C]-glucose injection. Boxplots show individual biological replicates; lines represent LOESS fits ± 95% CI. WT flies exhibit a time-structured labeling profile with an early-day rise in fractional labeling, whereas per mutants show attenuated temporal structure consistent with loss of circadian regulation of glucose handling and downstream carbon allocation. Raw data for these figures is available as S5 (A and B) and S6 (C) Data files. (PDF) [file pbio.3003717.s003.pdf]

**A**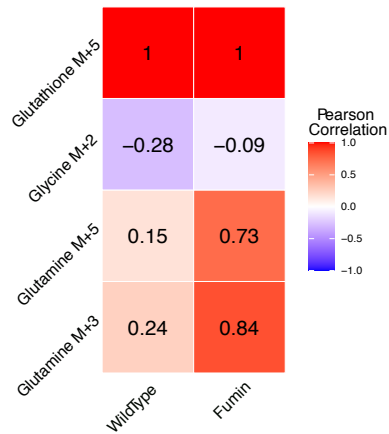**B**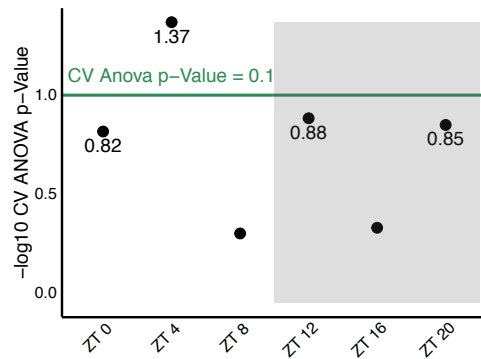**C**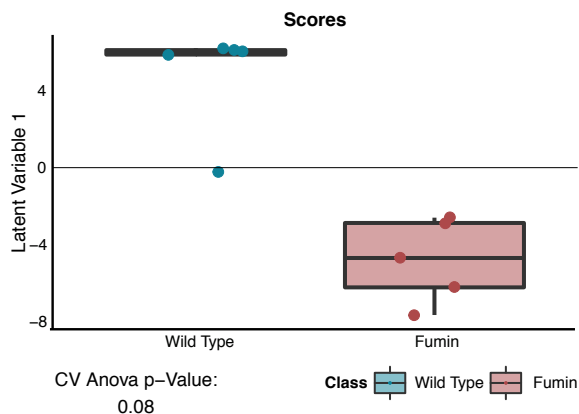**D**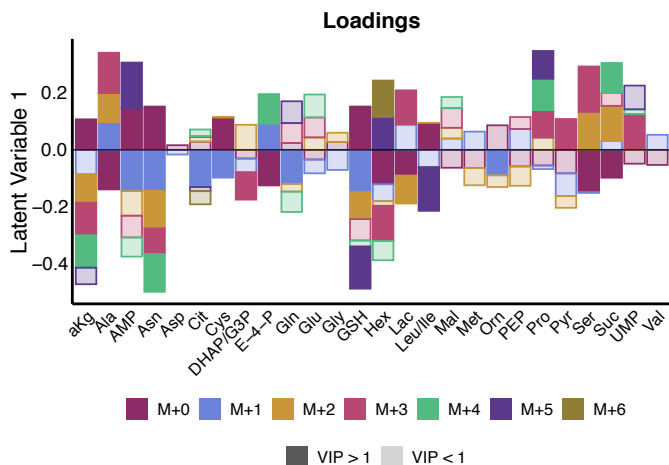

Supplement: S4 Fig — (A) Heatmap showing pearson correlation of select isotopologues with glutathione M + 5 for both wild type and fumin. (B) Negative log CV-ANOVA p-values for each time point tested in pairwise OPLS-DA models between genotypes. Significance was defined as a CV-ANOVA p-value less than 0.1 (shown as points above the green line). (C) Scores plot of the significant ZT 4 wild type versus fumin OPLS-DA model. Classes were defined as all ZT 4 samples for WT as one class and for fumin as a second class. (D) Loadings plot of the corresponding ZT 4 wild type versus fumin OPLS-DA model. Colors represent different isotopologues while shading representing VIP significance (dark signifies a VIP value greater than 1 while lighter shades are used for a VIP less than 1). Abbreviations are defined in Fig 3G. Raw data for these figures is available as S4 Data file. (PDF) [file pbio.3003717.s004.pdf]

**A****Scores**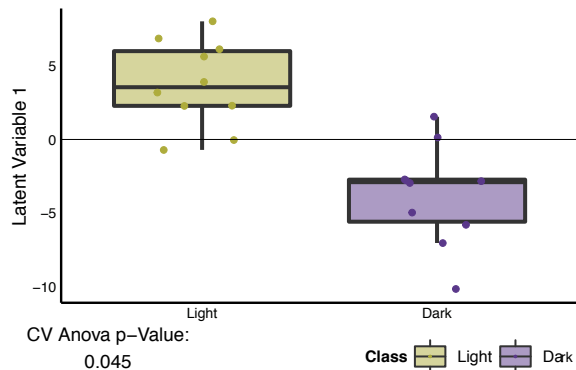**B****Loadings**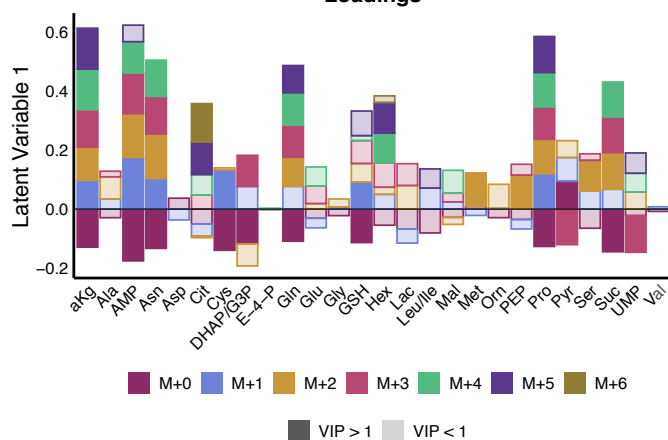**C**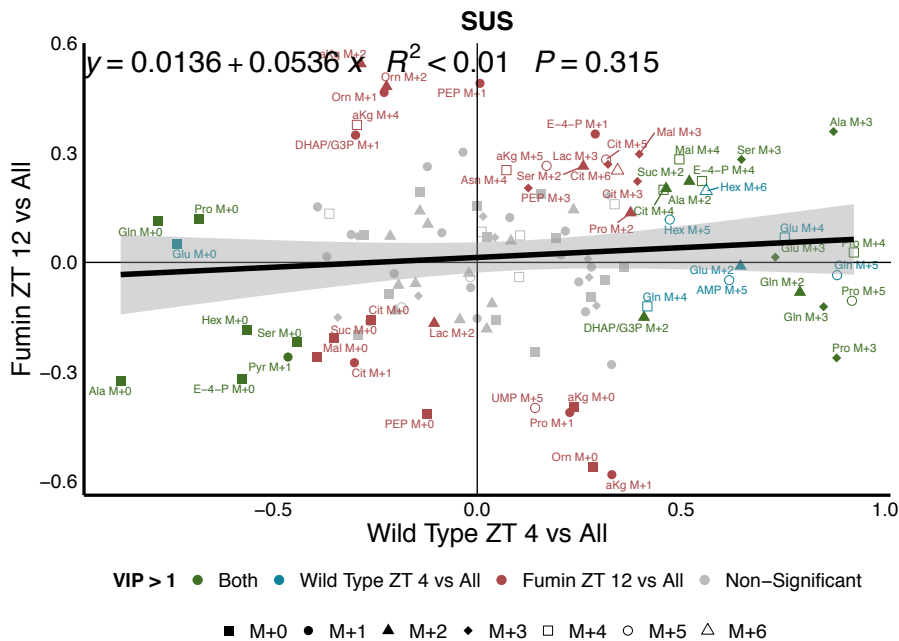

Supplement: S5 Fig — (A) Scores plot of the significant fumin Light (ZT 4 and ZT 8) versus Dark (ZT 16 and ZT 20) OPLS-DA model. (B) Corresponding loadings plot for the fumin Light versus Dark scores (A) plot. Colors represent different isotopologues and shading represents VIP significance. Abbreviations are defined in Fig 3G. (C) SUS plot comparing the loadings plot from the Wild Type ZT 4 versus All (Fig 3G) and fumin ZT 12 versus All (Fig 5E) OPLS-DA models with a regression line and equation. Green refers to VIP compounds in both models; blue refers to VIP compounds in the wild type model; red refers to VIP compounds in the fumin model. Shapes refer to isotopologues. Abbreviations are defined in Fig 3G. Raw data for these figures is available as S4 Data file. (PDF) [file pbio.3003717.s005.pdf]

Wild Type Fumin

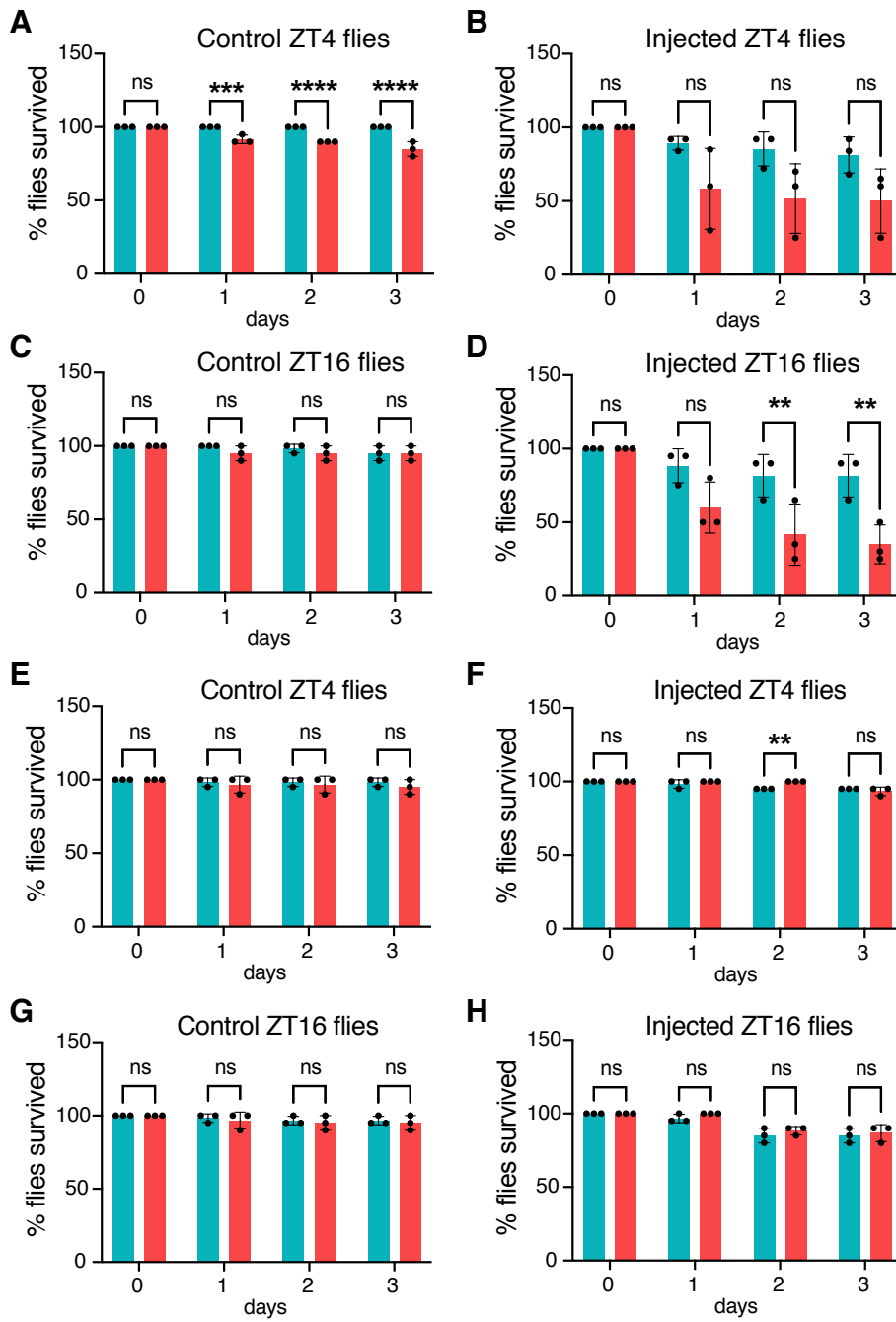

Supplement: S6 Fig — (A–D) Survival of wild-type and fumin flies fed 30 mM L-proline at ZT 4 (A, B) or ZT 16 (C, D) under control conditions (no injection) or following 12C-glucose injection. At ZT 4, proline feeding resulted in selective lethality of fumin flies under control conditions (A) but not after glucose injection (B). At ZT 16, fumin-specific lethality emerged only in the post-injection condition (D). (E–H) Survival of wild-type and fumin flies fed 15 mM L-proline at ZT 4 (E, F) or ZT 16 (G, H). Data represent N = 3 biological replicates (20–25 flies each replicate) per genotype per condition. Statistical analysis was performed using ANOVA Sidak’s multiple comparison test for obtaining P-values: *p < 0.05, **p < 0.005, and ***p < 0.0005. For all graphs, error bars = SEM and NS is not significant. Raw data for these figures is available as S7 Data file. (PDF) [file pbio.3003717.s006.pdf]

**A**

## Glycogen M+6 Enrichment

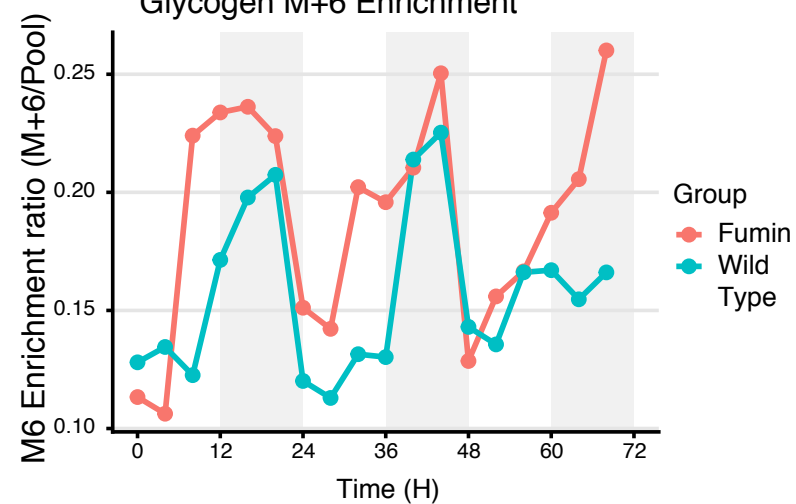**B**

Wild Type Fumin

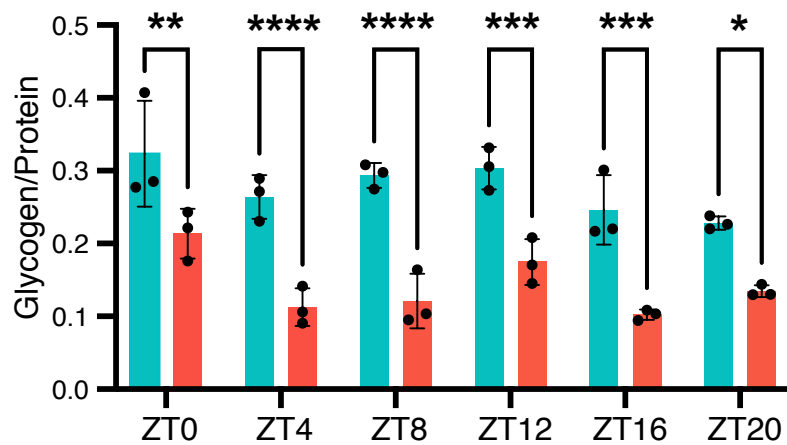**C**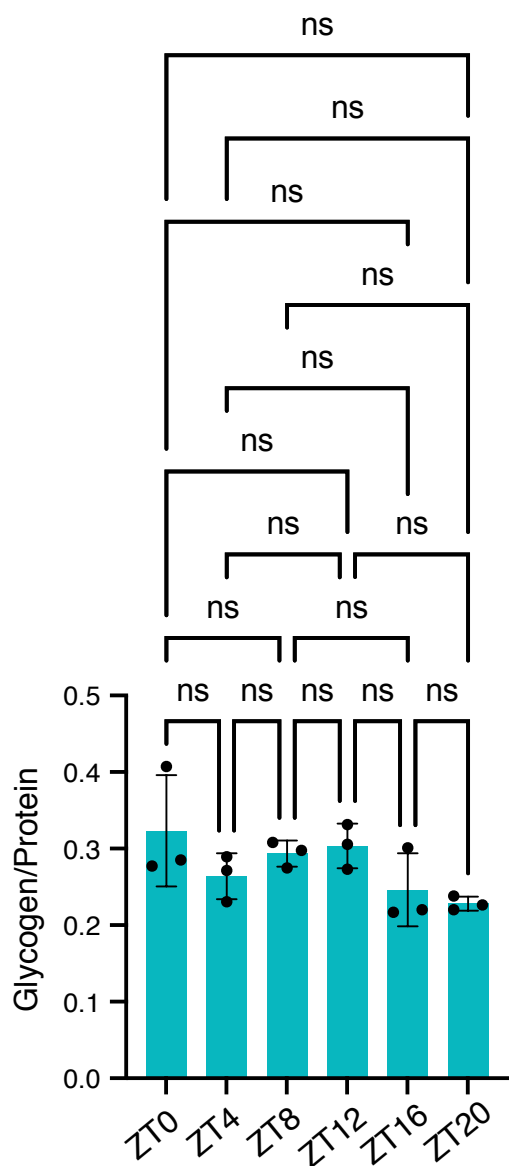**D**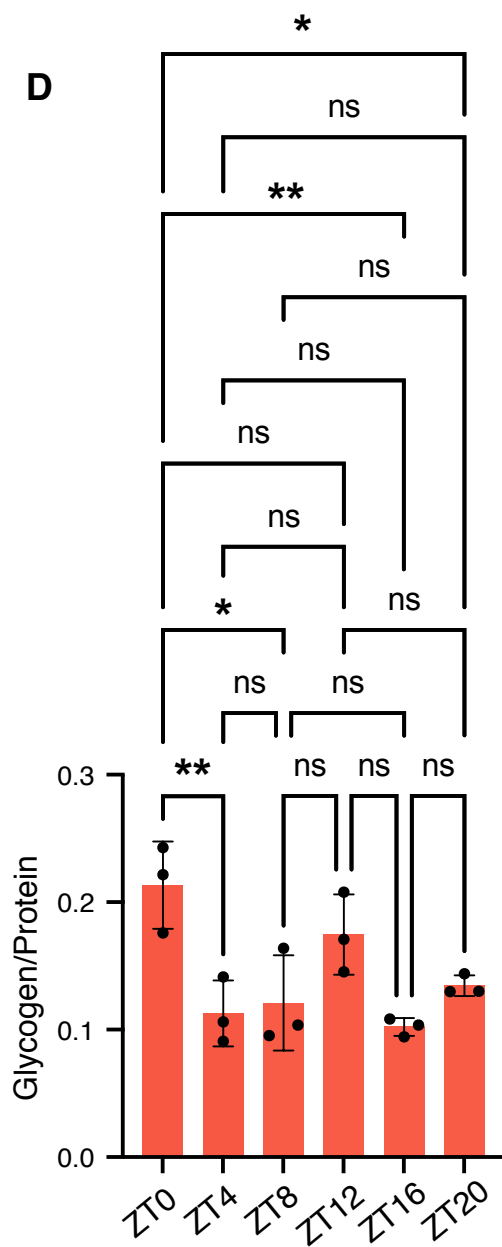

Supplement: S7 Fig — (A) Ratio of 13C-labeled hexose (M + 6) to total glycogen-derived glucose following enzymatic hydrolysis of whole-body glycogen in LD. Flies were injected with [U-13C]-glucose at each time point (n = 3/time point, shown as separate days), and glycogen-derived label was quantified by DESI–MS after hydrolysis. Shaded vertical bars denote subjective nighttime. (B) Quantification of glycogen levels in wild-type and fumin flies over a 24 hour light–dark cycle (ZT 0, 4, 8, 12, 16, 20). Glycogen levels were measured in 5-to 7-day—old males for both the genotypes. The resultant values were then normalized to protein content. Fly heads were removed prior to homogenization. N = 3 replicates. Each sample contained an independent group of 4 fly bodies. Within the mutant, glycogen showed a modest bimodal pattern, with ZT 0 and ZT 12 representing relative higher levels compared with other ZTs. Notably, these relative glycogen peaks in fumin did not coincide with the time window of maximal post-glucose-challenge flux, indicating that the shifted “rush hour” cannot be simply attributed to a change in glycogen synthesis or storage timing. Statistical analysis was performed using ANOVA Sidak’s multiple comparison test for obtaining P-values: *p < 0.05, **p < 0.005, and ***p < 0.0005 for B. For C and D, statistical analysis was performed using ANOVA Tukey’s multiple comparison test for obtaining P-values: *p < 0.05, **p < 0.005, and ***p < 0.0005. For all graphs, error bars = SEM and NS is not significant. Raw data for these figures is available as S8 (A) and S9 (B–D) Data files. (PDF) [file pbio.3003717.s007.pdf]

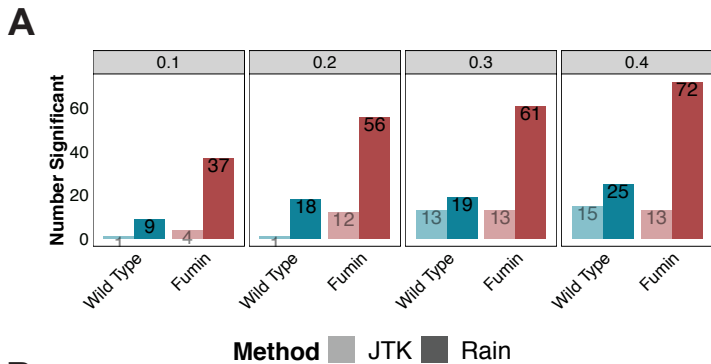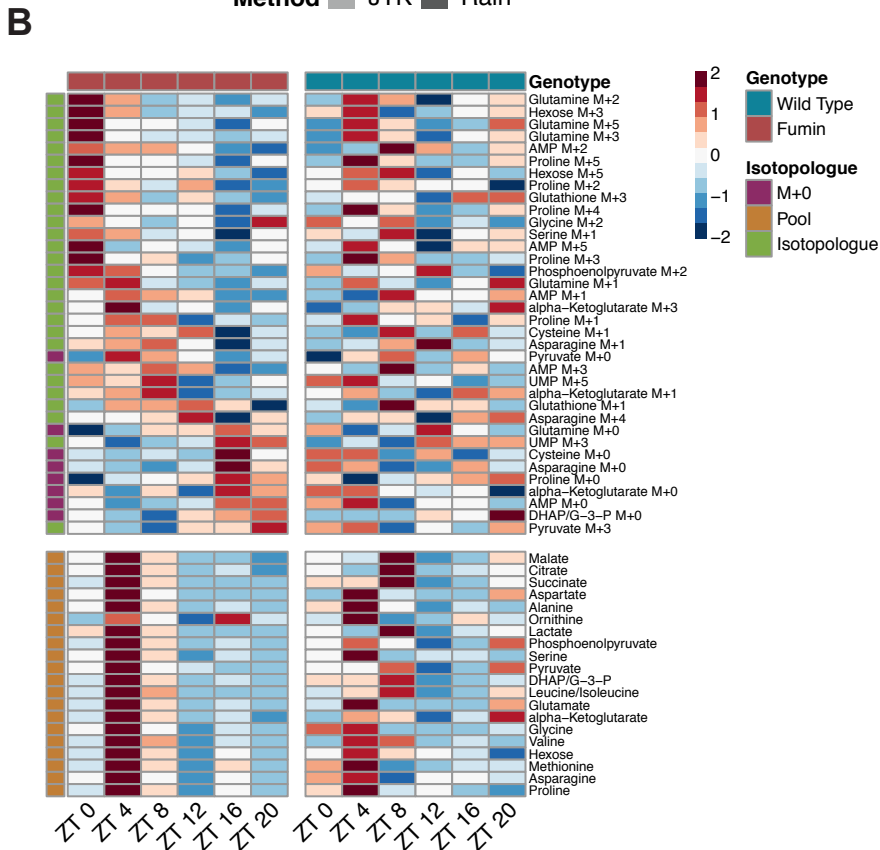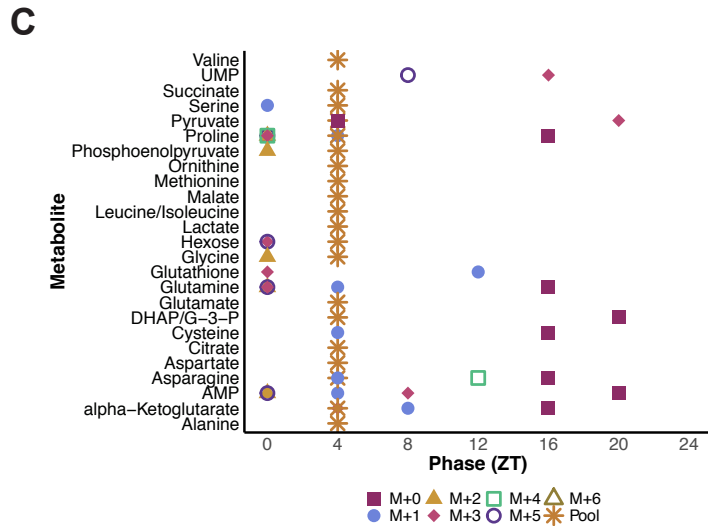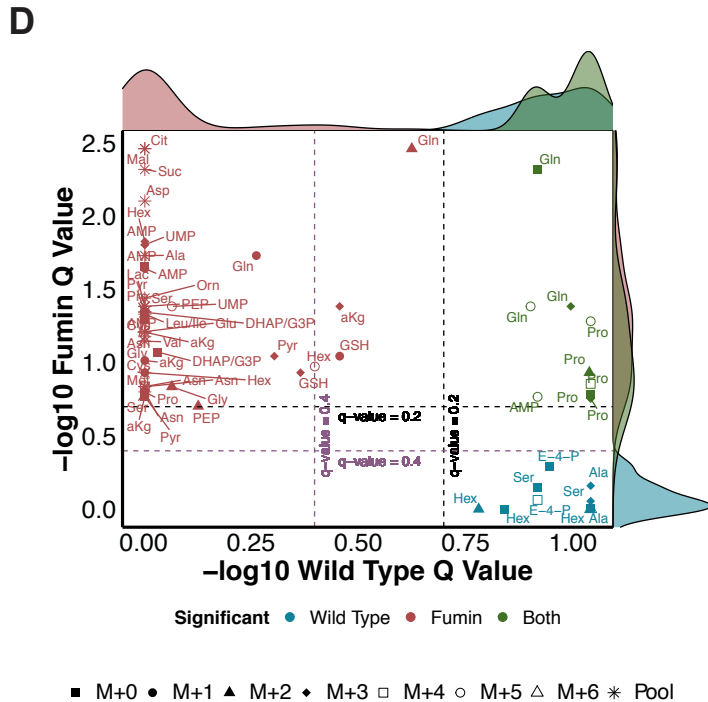

Supplement: S8 Fig — (A) Overview of the number of significant 20–28-hour rhythmic compounds observed at RAIN (dark) or JTK (light) q-value cut-offs of 0.1, 0.2, 0.3, and 0.4. (B) Phase-ordered heatmaps of significantly cycling compounds with periods of 20–28-hour in fumin at a RAIN q-value cutoff of 0.2. Enrichments for wild type are shown for comparison. (C) Distribution of RAIN phases for significant 20–28-hour compounds in fumin grouped by metabolite. Shapes and colors refer to isotopologues or pools. (D) Comparison of 20–28-hour significant cycling compounds in wild type and fumin as defined by a RAIN q-value less than 0.2. q-value cutoffs of 0.2 (black dotted line) and 0.4 (purple dotted line) are shown. Color refers to significant rhythmicity in one or both genotypes while shapes refer to isotopologues. Abbreviations are defined in Fig 3G. Raw data for these figures is available as S4 Data file. (PDF) [file pbio.3003717.s008.pdf]

A

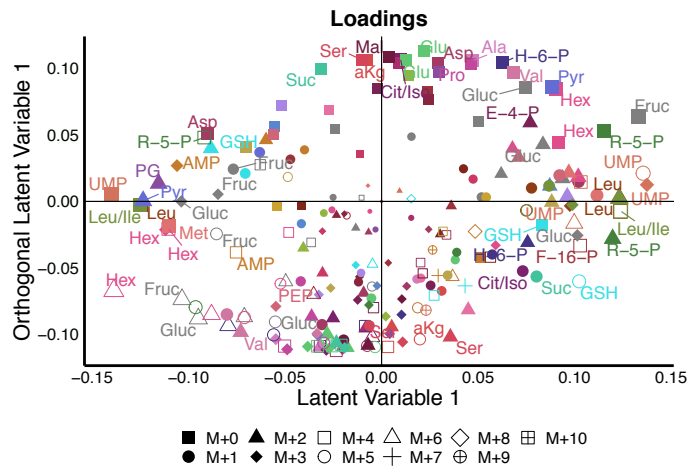

B

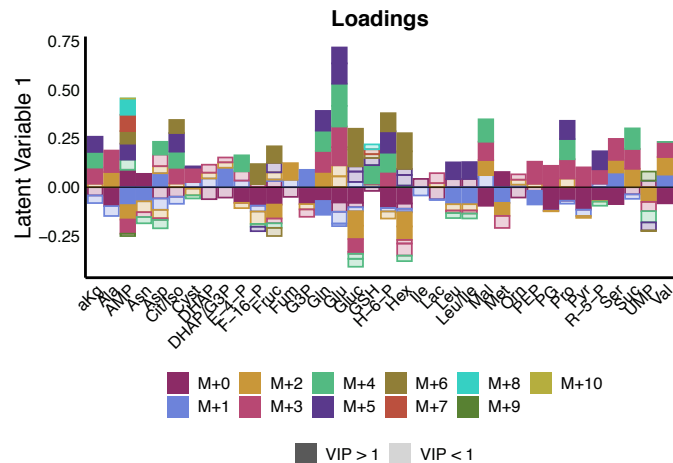

C

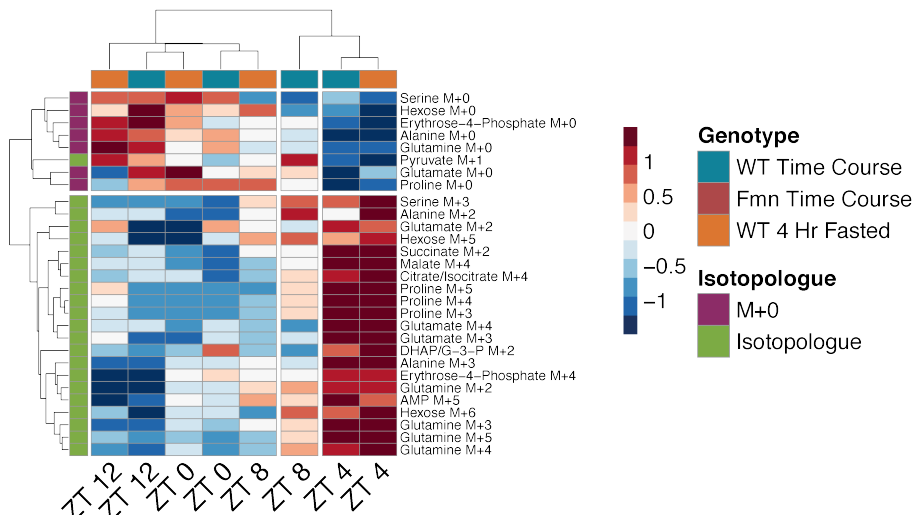

Supplement: S9 Fig — (A) Corresponding loadings plot for the ad libitum versus short-term fasted condition. Isotopologues are represented by shapes, metabolites by color and size of the shapes represents significance as defined through VIP values. Abbreviations as defined in Fig 3G; Isocitrate (Iso); Fructose-1,6-Phosphate (F-1,6-P); Fructose (Fruc); Fumarate (Fum); Glucose (Gluc); Hexose-6-Phosphate (H-6-P); Phosphoglycerates (PG); Ribulose-5-Phosphate (R-5-P). (B) Corresponding loadings plot for the short-term fasted ZT 4 versus All OPLS-DA model. Colors represent isotopologues with shading representing VIP significance (dark signifies a VIP value greater than 1 while lighter shades are used for a VIP less than 1). Abbreviations as defined in Fig 3G and 3A. (C) Clustered heatmap on both the x and y-axes shown using ZT 0–12 time points from the earlier Wild Type samples along with the short-term fasted samples for VIP compounds determined from the Wild Type ZT 4 versus All model (Fig 3F and 3G). Raw data for these figures is available as S4 and S10 Data files. (PDF) [file pbio.3003717.s009.pdf]
